# An Explainable Machine Learning Model for Early Prediction of Incident Myocardial Injury in Patients With Severe Fever With Thrombocytopenia Syndrome

**DOI:** 10.1002/jmv.71044

**Published:** 2026-06-29

**Authors:** Xiang Li, Xiaotong Yu, Wei Zhou, Sujuan Zhang, Zibo Fan, Yuanni Liu, Yi Shen, Zhenghua Zhao, Jianping Duan, Ling Lin, Zhihai Chen, Wei Zhang

**Affiliations:** ^1^ National Key Laboratory of Intelligent Tracking and Forecasting for Infectious Diseases, Beijing Ditan Hospital Capital Medical University Beijing P.R. China; ^2^ Dandong Infectious Disease Hospital Dandong P.R. China; ^3^ Dalian Public Health Clinical Center Dalian P.R. China; ^4^ Yantai Qishan Hospital Yantai P.R. China; ^5^ Taian City Central Hospital Taian P.R. China; ^6^ Qingdao Infectious Disease Hospital Qingdao P.R. China

**Keywords:** machine learning, myocardial injury, severe fever with thrombocytopenia syndrome, SHAP

## Abstract

This study aimed to develop an explainable machine learning–based model to enable early prediction of incident myocardial injury during hospitalization among patients with severe fever with thrombocytopenia syndrome (SFTS). This multicenter retrospective cohort study included and analyzed clinical data from 1,088 patients with SFTS who were hospitalized for the first time across four medical institutions between May 2011 and October 2024. The dataset was randomly split into an internal training set and a test set at a 7:3 ratio. After candidate predictors were selected from baseline clinical characteristics and laboratory indices, nine machine learning models were developed and compared, and external validation was conducted using retrospective cohorts from two independent medical centers. SHapley Additive Explanations (SHAP) were used to interpret model predictions and quantify feature importance. The ten key predictors identified comprised five baseline clinical characteristics and five laboratory indices. The CatBoost model demonstrated robust predictive performance in the internal test set and across two external validation cohorts, with AUCs of 0.750 (95% CI 0.698–0.803), 0.704 (95% CI 0.555–0.853), and 0.729 (95% CI 0.573–0.886), respectively. SHAP analysis further identified clinically relevant risk thresholds for the key laboratory predictors. In conclusion, this study developed a CatBoost‐based machine learning model that enables early prediction, at the time of hospital admission, of the risk of incident myocardial injury during hospitalization among patients with SFTS.

## 1 Introduction

Severe fever with thrombocytopenia syndrome (SFTS) is a tick‐borne infectious disease caused by *Bandavirus dabieense*, also known as SFTS virus (SFTSV) [1]. Since its first description in China in 2009, incidence has risen year on year across East Asia, with a reported case‐fatality rate of 12%–50% [2, 3, 4]. The clinical spectrum ranges from a mild illness characterized by fever, fatigue, leukopenia, and thrombocytopenia to a severe and potentially fatal course involving hemorrhage, neurological injury, myocardial injury, disseminated intravascular coagulation, and multiple organ failure [5, 6]. As a common complication of SFTS, myocardial injury is not only an important indicator of disease progression in affected patients [7], but has also been shown to be a key risk factor for fatal outcomes, as demonstrated by a prospective study conducted by Wang and colleagues [8]. Therefore, accurate early prediction of the risk of incident myocardial injury at hospital admission has important clinical implications for reducing mortality in patients with SFTS.

Currently, clinical assessment of myocardial injury in SFTS still relies heavily on myocardial enzyme biomarkers such as CK and CK‐MB, and existing studies have largely focused on predicting mortality. However, these biomarkers often become markedly abnormal only after organ injury has already occurred, making them insensitive to early pathophysiological changes and thereby limiting timely identification of high‐risk patients. Therefore, it is essential to develop predictive models that integrate multidimensional clinical information to enable early and individualized risk stratification. In recent years, machine learning (ML) has shown promising advantages in handling high‐dimensional, complex medical data and identifying nonlinear relationships, and has increasingly become an important methodological approach for developing clinical risk stratification tools [9, 10].

Against this background, this study conducted a multicenter retrospective cohort study of 1,088 patients with SFTS who were hospitalized for the first time at four institutions between 2011 and 2024. The study aimed to: (1) identify key admission‐time clinical features for early prediction of incident myocardial injury; (2) develop and validate ML‐based prediction models; (3) apply the SHAP algorithm to elucidate the model's decision logic, quantify the contribution of each variable, and determine clinically relevant risk thresholds for core laboratory predictors. Based on these findings, an online clinical prediction tool was further developed to support early risk assessment and individualized management of patients with SFTS.

## 2 Materials and Methods

### 2.1 Study Population

Between May 2011 and October 2024, 1,354 patients with a first hospital admission for SFTS were screened across four hospitals. After applying the eligibility criteria, 1,210 were included in the analysis. Data from 1,088 patients treated at Beijing Ditan Hospital, Dalian Public Health Clinical Center, Dandong Infectious Disease Hospital, and Yantai Infectious Disease Hospital were used for internal model development and testing. The remaining 122 patients—80 from Qingdao Infectious Disease Hospital and 42 from Taian City Central Hospital—constituted two independent external validation cohorts; the two centers were analyzed separately to limit the influence of inter‐center heterogeneity on validation estimates.

The inclusion criteria were as follows: (1) age ≥ 18 years; (2) laboratory‐confirmed infection with SFTS virus, defined as positive virus isolation, positive SFTSV nucleic acid testing, or a ≥ 4‐fold rise in antibody titers between paired serum samples [11]. The exclusion criteria were: (1) myocardial injury present at admission. Myocardial injury was defined as a serum cardiac troponin I (cTnI) level above the 99th percentile upper reference limit [12] (0.028 ng/mL, based on the laboratory reference standard of Beijing Ditan Hospital). Serum cTnI was measured using a Mindray CL‐8000i automated chemiluminescence immunoassay analyzer in combination with the Mindray high‐sensitivity cardiac troponin I (hs‐cTnI) assay kit (chemiluminescence immunoassay; Shenzhen Mindray Bio‐Medical Electronics Co. Ltd., Shenzhen, China); (2) incomplete clinical data, with missing data accounting for > 5% of the total medical record; (3) pre‐existing comorbidities, including coronary artery disease, chronic liver disease, end‐stage renal disease, hematologic disorders, autoimmune diseases, or malignancy. The external validation cohorts applied the same inclusion and exclusion criteria as the primary cohort.

The clinical investigation was conducted in strict accordance with the ethical principles outlined in the Declaration of Helsinki. The study protocol received formal ethical approval from the Institutional Review Board of Beijing Ditan Hospital, Capital Medical University (No. DTEC‐KY2022‐022‐02). Given the retrospective nature of this study and the use of de‐identified, routinely collected clinical data, an application for a waiver of informed consent was submitted to and subsequently granted by the Institutional Review Board of Beijing Ditan Hospital. All patient data were processed and analyzed in an anonymized manner to ensure confidentiality.

### 2.2 Data Collection and Variables

All data were extracted from the hospitals' electronic medical record systems, including demographic information (age, sex, comorbidities), clinical symptoms and signs (fever, fatigue, headache, bulbar conjunctival edema, pharyngeal swelling, and others), and laboratory test results, including white blood cell count (WBC), neutrophil count (NEUT#), lymphocyte count (LYMPH#), monocyte count (MONO#), eosinophil count (EO#), basophil count (BASO#), red blood cell count (RBC), hemoglobin (Hb), mean corpuscular volume (MCV), mean corpuscular hemoglobin concentration (MCHC), red cell distribution width (RDW), platelet count (PLT), mean platelet volume (MPV), serum potassium (K), serum sodium (Na), serum chloride (Cl), serum calcium (Ca), blood urea nitrogen (BUN), creatinine (Cr), prothrombin time (PT), prothrombin activity (PTA), activated partial thromboplastin time (APTT), thrombin time (TT), alanine aminotransferase (ALT), aspartate aminotransferase (AST), total bilirubin (TBIL), direct bilirubin (DBIL), total protein (TP), albumin (ALB), globulin (GLB), gamma‐glutamyl transferase (GGT), alkaline phosphatase (ALP), and cholinesterase (CHE). To evaluate the predictive value of non‐specific indicators for myocardial injury and to avoid circular reasoning, this study did not include direct biomarkers of myocardial injury—such as lactate dehydrogenase (LDH), creatine kinase (CK), or creatine kinase‐MB (CK‐MB)—in the data analysis and model development, and no derived features were generated.

### 2.3 Data Preprocessing

A uniform preprocessing pipeline was applied to the internal cohort and both external validation cohorts. During data cleaning, records with missing identifiers and empty rows were removed, and values outside clinically plausible ranges were excluded: age 18–100 years, K 1.0–10.0 mmol/L, Na 100–200 mmol/L, WBC < 500 × 10^9^/L, and PLT < 2000 × 10^9^/L. Second, regarding missing data, variables with a missing rate exceeding 30% in the internal cohort were predefined for exclusion; however, all included variables fell below this threshold (details are provided in Supporting Table 17), and no variable was ultimately removed on this basis. For the remaining missing data, a tiered imputation strategy was applied: variables with < 5% missingness were imputed using the median (continuous) or mode (categorical), whereas those with 5%–30% missingness were handled by multiple imputation by chained equations (MICE; m = 5), with logistic regression for binary features. The external validation cohorts were processed according to the preprocessing workflow used for the internal development cohort, including feature alignment and standardization. For missing data, this study referred to the recommendation by Sisk et al. (2023) [13] that the handling of missing data during prediction model validation should, as far as possible, mimic the strategy intended for actual deployment. Accordingly, missing values in the external cohorts were imputed using the imputation parameters determined during the internal training stage, without including the outcome variable in the imputation process, to simulate the clinical deployment setting in which the model is applied to new patients. In addition, a sensitivity analysis was further conducted using summary statistics from each external cohort, with continuous variables imputed by the median and binary variables by the mode, while keeping the model parameters and the optimal decision threshold determined from the internal training set unchanged.

### 2.4 Feature Selection and Collinearity Control

A tiered strategy combining multi‐algorithm screening and stability assessment was employed. Within each of the five imputed datasets, preliminary feature screening was performed using LASSO regression (10‐fold cross‐validation, λ selected by the λmin criterion) and the Boruta algorithm (500 iterations). Stable predictors were identified through 1,000 bootstrap resamples, retaining variables with a selection frequency ≥ 80%. Redundant variables with Pearson correlation coefficients > 0.85 were removed to control multicollinearity. Regression estimates across the five datasets were then pooled using Rubin's rules; variables selected in ≥ 4 datasets with a pooled *p* < 0.05 were retained. In addition, although age showed a relatively low selection frequency (2/5), it was manually retained in the final model as a baseline adjustment variable given its well‐established prognostic relevance in SFTS [14, 15].

### 2.5 Model Development

This study developed prediction models using multiple machine learning algorithms, including Logistic Regression (Logistic), Support Vector Machine (SVM), Gradient Boosting Machine (GBM), Artificial Neural Network (Neural Network), Random Forest, K Nearest Neighbors (KNN), Adaptive Boosting (AdaBoost), Light Gradient Boosting Machine (LightGBM), and Categorical Boosting (CatBoost).

The dataset was randomly split into a training set (70%) and a test set (30%) using stratified sampling to preserve the outcome distribution. Model hyperparameters were tuned via grid search, and repeated 10‐fold cross‐validation (5 repeats) was performed to ensure robustness. The optimized hyperparameters for each model are provided in Supporting Table 1.

To address class imbalance, the default probability cutoff of 0.5 was not used. Instead, threshold optimization was performed by plotting receiver operating characteristic (ROC) curves and applying the Youden index to determine the optimal probability threshold in the training set, thereby balancing sensitivity and specificity. Model performance was comprehensively evaluated using the area under the ROC curve (AUC), accuracy, sensitivity, specificity, and F1 score, and clinical utility was further assessed using decision curve analysis (DCA). Finally, SHAP was used to interpret feature importance and quantify the contribution of individual predictors.

## 3 Statistical Analysis

Non‐normally distributed continuous variables are presented as median (interquartile range) and compared using the Wilcoxon rank‐sum test. Categorical variables are presented as counts (percentages) and compared using the chi‐square or Fisher's exact test, as appropriate. All analyses were conducted in R (version 4.4.3), and a two‐sided *p* < 0.05 was considered statistically significant.

## 4 Results

### 4.1 Baseline Characteristics of the Internally Modeled Cohort

**Figure 1:**
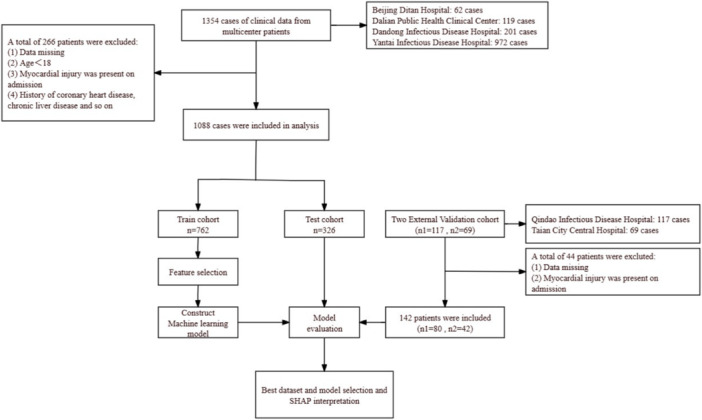
Flowchart of patient selection and study design.

A total of 1,088 laboratory‐confirmed SFTS patients admitted for the first time were included in the internal cohort and classified according to whether myocardial injury occurred during hospitalization: 607 (55.8%) in the myocardial injury group and 481 (44.2%) in the non–myocardial injury group. The study flow diagram is shown in Figure 1.

In the internal cohort, the overall median age was 64 years. Patients in the myocardial injury group were significantly older (66 vs. 62 years, *p* < 0.001) and included a higher proportion of women (55.4% vs. 43.7%, *p* < 0.001). Hypertension (20.3% vs. 11.6%, *p* < 0.001) and diabetes (10.2% vs. 4.2%, *p* < 0.001) were more prevalent in the myocardial injury group. Clinically, patients with myocardial injury exhibited more pronounced systemic compromise, with higher frequencies of fatigue (79.7% vs. 69.3%), lethargy (54.1% vs. 44.4%), and vomiting (22.5% vs. 15.6%; all *p* < 0.05). Conversely, several localized and systemic manifestations of SFTS and signs suggestive of microcirculatory disturbance were more common in the non–myocardial injury group, including headache (28.8% vs. 10.0%), pharyngeal swelling (19.7% vs. 6.7%), bulbar conjunctival edema (10.6% vs. 3.6%), cutaneous congestion (11.7% vs. 3.8%), rash (6.2% vs. 3.4%), and bilateral renal percussion tenderness (8.2% vs. 0.5%; all *p* < 0.05). Laboratory findings in the myocardial injury group showed more prominent biochemical and coagulation disturbances, characterized by higher GLB, PTA, and APTT, and significantly lower ALB, Ca, WBC, and LYMPH# (all *p* < 0.05). Table 1.

**Table 1:** Baseline characteristics comparison of Internal training cohort between myocardial injury and non‐myocardial injury.

Characteristics	Overall (*N*＝1088)	Non‐myocardial‐injury (*N*＝481)	Myocardial‐injury (*N*＝607)	*p* value
**General information, median (IQR) or *n* (%)**				
Age (years)	64.00 [56.00, 71.00]	62.00 [53.00, 70.00]	66.00 [58.00, 73.00]	< 0.001
Gender				< 0.001
Female	546 (50.2)	210 (43.7)	336 (55.4)	
Male	542 (49.8)	271 (56.3)	271 (44.6)	
**History of underlying disease, *n* (%)**				
Hypertension	179 (16.5)	56 (11.6)	123 (20.3)	< 0.001
Diabetes	82 (7.5)	20 (4.2)	62 (10.2)	< 0.001
Cerebral infarction	37 (3.4)	18 (3.7)	19 (3.1)	0.7
**Symptoms at admission, *n* (%)**				
Fever	885 (81.3)	379 (78.8)	506 (83.4)	0.065
Fatigue	771 (75.2)	305 (69.3)	466 (79.7)	< 0.001
Lethargy	511 (50.0)	196 (44.4)	315 (54.1)	0.003
Headache	193 (18.4)	135 (28.8)	58 (10.0)	< 0.001
Palpitation	18 (1.7)	15 (3.2)	3 (0.5)	0.002
Muscle soreness	402 (38.3)	190 (40.6)	212 (36.5)	0.195
Arthralgia	251 (24.0)	122 (26.2)	129 (22.2)	0.159
Anorexia	814 (76.6)	355 (74.4)	459 (78.3)	0.155
Nausea	467 (44.1)	228 (48.1)	239 (40.9)	0.023
Vomiting	199 (19.5)	68 (15.6)	131 (22.5)	0.008
Abdominal pain	114 (10.8)	66 (14.1)	48 (8.2)	0.003
Diarrhea	235 (22.4)	97 (20.6)	138 (23.8)	0.258
Melena	18 (1.7)	10 (2.1)	8 (1.4)	0.483
Cough	162 (15.4)	80 (17.0)	82 (14.1)	0.222
Oliguria	44 (4.2)	34 (7.2)	10 (1.7)	< 0.001
Cutaneous congestion	77 (7.3)	55 (11.7)	22 (3.8)	< 0.001
Rash	49 (4.7)	29 (6.2)	20 (3.4)	0.047
Petechia	87 (8.4)	40 (8.7)	47 (8.1)	0.848
Pharyngeal swelling	131 (12.5)	92 (19.7)	39 (6.7)	< 0.001
Bulbar conjunctival edema	65 (6.5)	44 (10.6)	21 (3.6)	< 0.001
Lymphadenopathy	200 (19.0)	92 (19.7)	108 (18.5)	0.699
Hemorrhage	21 (2.0)	13 (2.8)	8 (1.4)	0.159
Bilateral renal percussion tenderness	41 (3.9)	38 (8.2)	3 (0.5)	< 0.001
**Laboratory variables, median (IQR)**				
WBC (10^9/L)	2.28 [1.51, 3.90]	2.43 [1.60, 4.34]	2.13 [1.46, 3.50]	0.001
NEUT# (10^9/L)	1.32 [0.83, 2.44]	1.38 [0.83, 2.76]	1.29 [0.84, 2.18]	0.192
LYMPH# (10^9/L)	0.58 [0.38, 0.97]	0.68 [0.41, 1.17]	0.51 [0.34, 0.82]	< 0.001
MONO# (10^9/L)	0.16 [0.08, 0.40]	0.18 [0.08, 0.43]	0.15 [0.08, 0.37]	0.246
EO# (10^9/L)	0.00 [0.00, 0.01]	0.00 [0.00, 0.01]	0.00 [0.00, 0.01]	0.741
BASO# (10^9/L)	0.00 [0.00, 0.01]	0.01 [0.00, 0.02]	0.00 [0.00, 0.01]	0.05
RBC (10^12/L)	4.54 [4.23, 4.94]	4.55 [4.23, 4.93]	4.54 [4.21, 4.95]	0.957
Hb (g/L)	138.00 [128.00, 151.00]	139.00 [128.00, 151.00]	138.00 [128.00,151.00]	0.893
MCV (fl)	88.80 [85.50, 92.20]	89.00 [85.40, 92.70]	88.60 [85.62, 91.80]	0.358
MCHC (g/L)	341.00 [332.00, 350.00]	340.00 [332.00, 350.00]	342.00 [332.00, 351.00]	0.11
RDW (%)	12.70 [12.15, 13.60]	13.10 [12.30, 14.20]	12.50 [12.00, 13.30]	< 0.001
PLT (10^9/L)	61.00 [44.00, 82.00]	58.00 [40.00, 83.00]	62.00 [48.00, 82.00]	0.038
MPV (fl)	10.60 [9.90, 11.40]	10.90 [10.00, 11.80]	10.50 [9.80, 11.20]	< 0.001
K (mmol/L)	3.80 [3.50, 4.10]	3.80 [3.50, 4.15]	3.80 [3.49, 4.10]	0.158
Na (mmol/L)	135.00 [132.00, 138.00]	134.50 [131.00, 137.90]	135.70 [132.20, 138.00]	0.008
Cl (mmol/L)	98.80 [95.80, 102.00]	99.00 [96.00, 102.00]	98.22 [95.00, 101.53]	0.041
Ca (mmol/L)	1.94 [1.84, 2.04]	1.97 [1.87, 2.07]	1.92 [1.82, 2.02]	< 0.001
BUN (mmol/L)	5.40 [3.99, 7.54]	5.26 [4.01, 7.47]	5.51 [3.96, 7.64]	0.938
Cr (μmol/L)	65.20 [53.59, 80.62]	67.00 [55.00, 83.00]	65.00 [52.11, 78.28]	0.018
PT (s)	12.50 [11.70, 13.30]	12.40 [11.50, 13.30]	12.55 [11.80, 13.30]	0.156
PTA (%)	107.00 [92.40, 122.00]	103.00 [87.25, 122.10]	108.00 [96.00, 121.75]	0.004
APTT (s)	44.40 [37.60, 51.58]	41.30 [34.85, 49.75]	46.20 [39.60, 52.50]	< 0.001
TT (s)	20.10 [17.70, 22.80]	19.30 [16.70, 21.30]	20.80 [18.22, 24.00]	< 0.001
ALT (U/L)	72.00 [41.50, 137.90]	69.20 [39.00, 128.20]	74.30 [44.15, 145.10]	0.179
AST (U/L)	110.35 [56.98, 252.00]	108.25 [55.00, 257.67]	112.25 [58.00, 238.00]	0.768
TBIL (μmol/L)	9.70 [7.48, 13.30]	9.86 [7.20, 13.80]	9.65 [7.69, 13.00]	0.81
DBIL (μmol/L)	3.80 [2.50, 5.71]	4.00 [2.68, 5.89]	3.62 [2.41, 5.60]	0.07
TP (g/L)	58.00 [53.90, 62.40]	58.00 [53.58, 62.60]	58.05 [54.20, 62.10]	0.955
ALB (g/L)	32.70 [29.40, 35.79]	33.70 [30.30, 36.58]	31.98 [28.90, 35.25]	< 0.001
GLB (g/L)	25.40 [22.64, 28.39]	24.40 [21.80, 27.80]	25.96 [23.20, 28.70]	< 0.001
GGT (U/L)	32.00 [20.00, 69.50]	37.15 [22.38, 88.42]	29.00 [19.00,57.00]	< 0.001
ALP (U/L)	62.90 [51.00, 80.30]	66.00 [52.00, 85.00]	61.00 [50.77,79.00]	0.009
CHE (U/L)	5973.00 [4902.97, 7071.74]	6126.00 [4969.00,7300.00]	5820.94 [4867.98,6909.50]	0.032

*Note:* Continuous variables with non‐normal distribution are reported as median (interquartile range, IQR) and compared using the Wilcoxon rank‐sum test. Categorical variables are expressed as *n* (%) and compared using the Chi‐square test or Fisher's exact test, as appropriate.

Abbreviations: ALB, albumin; ALP, alkaline phosphatase; ALT, alanine aminotransferase; APTT, activated partial thromboplastin time; AST, aspartate aminotransferase; BASO#, Absolute Basophil Count; BUN, blood urea nitrogen; Ca, serum calcium; CHE, cholinesterase; Cl, serum chloride; Cr, Creatinine; DBIL, direct bilirubin; EO#, Absolute Eosinophil Count; GGT, gamma‐glutamyl transferase; GLB, Globulin; Hb, hemoglobin; K, serum potassium; LYMPH#, Absolute Lymphocyte Count; MCHC, mean corpuscular hemoglobin concentration; MCV, mean corpuscular volume; MONO#, Absolute Monocyte Count; MPV, mean platelet volume; Na serum sodium; PLT, platelet count; PT, prothrombin time; PTA, prothrombin activity; RBC, red blood cell count; RDW, red cell distribution width; SFTS, severe fever with thrombocytopenia syndrome, NEUT#, Absolute Neutrophil Count; TBIL, total bilirubin; TP, total protein; TT, thrombin time; WBC, white blood cell count.

### 4.2 External Validation Characteristics of the Study Cohort

In the Qingdao cohort (*n* = 80), no significant differences were observed between groups in age, sex, or comorbidities (all *p* > 0.05). The myocardial injury group had a higher frequency of muscle soreness (50.0% vs. 21.2%, *p* < 0.05) but a lower incidence of pharyngeal swelling (15.0% vs. 51.8%, *p* < 0.05). Lymphocyte count, serum calcium, and albumin were significantly lower in the myocardial injury group (all *p* < 0.05). Other baseline characteristics did not differ significantly. Details are provided in Supporting Table 2.

In the Taian cohort (*n* = 42), only serum calcium and albumin were lower in the myocardial injury group (all *p* < 0.01); no other baseline characteristics differed significantly. Details are provided in Supporting Table 3.

### 4.3 Feature Selection

**Figure 2:**
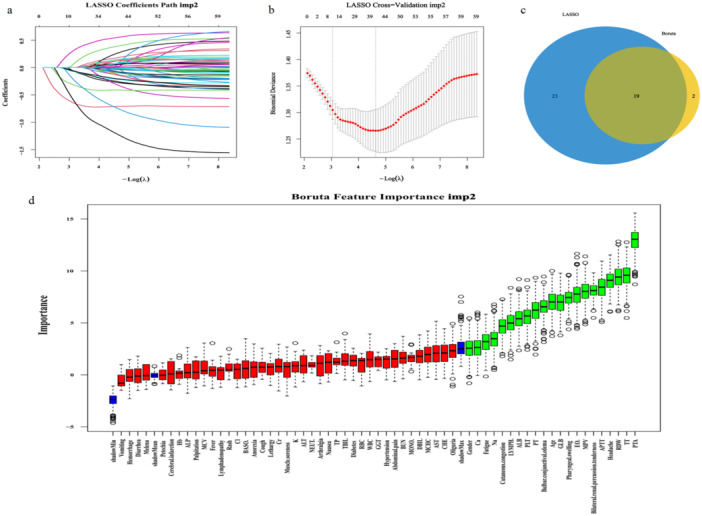
Feature selection by LASSO regression and Boruta algorithm. (a) LASSO coefficient paths showing variable shrinkage as λ increases. (b) Cross‐validation plot; dashed lines indicate λ_min (left) and λ_1se (right). (c) Venn diagram showing 19 common features selected by both LASSO (23 total) and Boruta (21 total). (d) Boruta importance plot; green boxes indicate confirmed important features, red boxes indicate rejected features. LASSO Least Absolute Shrinkage and Selection Operator.

To identify potential predictors of incident myocardial injury, this study first performed preliminary feature screening in each of the five multiply imputed datasets using LASSO regression (λ selected by the λmin criterion based on 10‐fold cross‐validation) and the Boruta algorithm (500 iterations). LASSO regression was implemented using the “glmnet” package in R (version 4.1‐10). Boruta, a random forest (RF)–based wrapper method, was performed using the R package “Boruta” (version 9.0.0). The two approaches yielded highly concordant results, which were visualized as Venn diagrams (Figure 2 shows results for the optimal imputed dataset 2). Redundant variables with |r | > 0.85 were removed to control multicollinearity, and stability validation through 1,000 bootstrap resamples retained only variables with selection frequency ≥ 80%. Robust predictors from each imputed dataset were entered into multivariable logistic regression, and results were pooled using Rubin's rules (Table 2); variables selected in ≥ 4 datasets with pooled *p* < 0.05 were retained.

**Table 2:** Pooled multivariable logistic regression analysis of candidate predictors for new‐onset myocardial injury.

term	Frequency	Pooled OR (95% CI)	Final *p* value
Fatigue	5	1.907 (1.286–2.829)	0.001
LYMPH#	5	0.729 (0.591–0.899)	0.003
ALB	5	0.764 (0.626–0.932)	0.008
Bulbar conjunctival edema	5	0.373 (0.179–0.777)	0.008
Pharyngeal swelling	5	0.506 (0.268–0.954)	0.035
Diabetes	5	2.009 (0.997–4.045)	0.051
Headache	5	0.383 (0.235–0.626)	< 0.001
K	4	0.812 (0.670–0.985)	0.035
GLB	4	1.228 (1.013–1.490)	0.037
Ca	4	0.782 (0.614–0.995)	0.046
ALP	4	0.581 (0.319–1.060)	0.077
Nausea	3	0.548 (0.365–0.822)	0.004
RBC	3	1.324 (1.090–1.608)	0.005
Na	3	1.282 (1.049–1.567)	0.015
Vomiting	3	1.773 (1.078–2.917)	0.024
Gender	3	0.651 (0.446–0.950)	0.026
TT	3	1.176 (0.859–1.610)	0.312
ALT	2	1.279 (1.054–1.552)	0.013
RDW	2	0.850 (0.714–1.012)	0.069
GGT	2	0.848 (0.697–1.031)	0.098
MCHC	2	1.965 (0.856–4.509)	0.111
Age	2	1.138 (0.946–1.368)	0.169
PT	1	0.751 (0.617–0.914)	0.004
APTT	1	1.236 (1.012–1.509)	0.037
BUN	1	0.749 (0.498–1.126)	0.165

*Note:* Frequency indicates the number of imputed datasets (out of 5) in which each variable was selected. Pooled OR and 95% CI were calculated using Rubin's rules. Variables with Frequency ≥ 4 and pooled *p* < 0.05 were retained for final model construction. Age was manually retained due to its established clinical significance in SFTS prognosis despite a lower selection frequency (2/5).

Abbreviations: CI, confidence interval; OR, odds ratio.

Following the above procedures, ten core predictors were ultimately identified: fatigue, headache, pharyngeal swelling, bulbar conjunctival edema, lymphocyte count, albumin, globulin, serum calcium, serum potassium, and age.

### 4.4 Construction and Evaluation of Machine Learning Models

Based on the internal training set (*n* = 762), internal test set (*n* = 326), and two external validation cohorts (*n*1 = 80 and *n*2 = 42), this study evaluated the predictive performance of the nine machine learning models described above.

**Figure 3:**
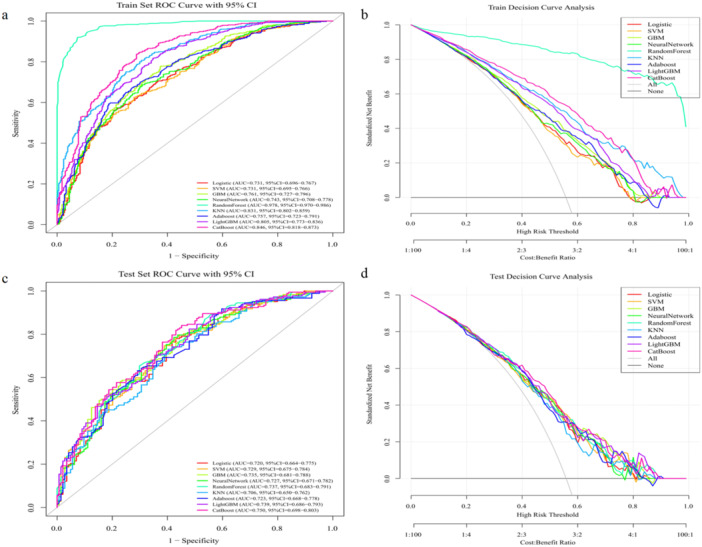
Performance evaluation of machine learning models in the internal cohort. (a) ROC curves of nine models in the training set with AUC and 95% CI. (b) Decision curve analysis (DCA) in the training set; gray line (“All”) represents treating all patients, black line (“None”) represents treating no patients. (c) ROC curves in the internal test set. (d) DCA in the internal test set. ROC Receiver Operating Characteristic, AUC Area Under the Curve, CI Confidence Interval, DCA Decision Curve Analysis.

**Figure 4:**
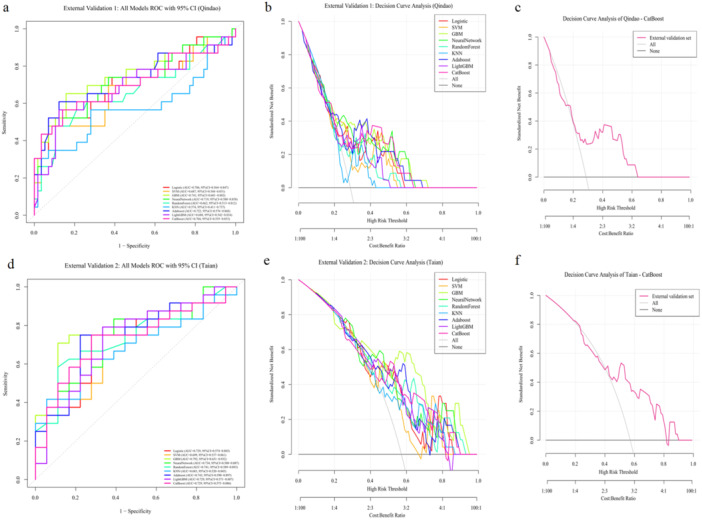
Performance evaluation of machine learning models in the external validation cohorts. (a) ROC curves of nine models in Qingdao cohort (*n* = 80) with AUC and 95% CI. (b) DCA of all models in Qingdao cohort. (c) DCA of CatBoost model in Qingdao cohort. (d) ROC curves in Taian cohort (*n* = 42). (e) DCA of all models in Taian cohort. (f) DCA of CatBoost model in Taian cohort.

First, the overall performance of the nine machine learning algorithms was assessed across the five imputed datasets. To minimize the influence of probability‐threshold selection on model comparison, the threshold‐independent metric—area under the ROC curve (AUC)—was used as the primary criterion for selecting a representative imputed dataset. The analysis revealed that Imp2 not only achieved the highest AUC in the internal test set (0.730) but also showed a relatively smaller AUC gap between the training and test sets (0.068), suggesting a lower risk of overfitting in this imputed dataset. Therefore, Imp2 was chosen as the representative dataset for subsequent optimal model selection and interpretation. Based on Imp2, model performance metrics were further compared in detail (Tables 3 and 4 for the training and test sets, respectively; Supporting Tables 4 and 5 for External Validation Cohorts 1 and 2, respectively; and Figures 3 and 4). The model performance comparisons between training and validation sets for the remaining four datasets (imp1 and imp3–5) are presented in Supporting Tables 7–14.

**Table 3:** Performance comparison of different machine learning models in the training set imp2.

Model	AUC	Threshold	Accuracy	Sensitivity	Specificity	F1 Score
Logistic	0.731	0.648	0.664	0.562	0.792	0.651
SVM	0.731	0.585	0.668	0.626	0.721	0.678
GBM	0.761	0.543	0.71	0.776	0.626	0.749
Neural Network	0.743	0.559	0.689	0.687	0.691	0.711
Random Forest	0.978	0.57	0.923	0.92	0.926	0.93
KNN	0.831	0.535	0.757	0.807	0.694	0.788
Adaboost	0.757	0.62	0.69	0.593	0.813	0.681
LightGBM	0.805	0.552	0.744	0.816	0.653	0.781
CatBoost	0.846	0.527	0.782	0.838	0.712	0.811

Abbreviations: AdaBoost, adaptive boosting; CatBoost, categorical boosting; GBM, gradient boosting machine; LightGBM, light gradient boosting machine; KNN, K nearest neighbors; LLR, logistic logistic regression; Neural Network Artificial Neural Network; SVM, support vector machine.

**Table 4:** Performance comparison of different machine learning models in the test set imp2.

Model	AUC	Threshold	Accuracy	Sensitivity	Specificity	F1 score
Logistic	0.72	0.527	0.687	0.78	0.569	0.736
SVM	0.729	0.499	0.687	0.747	0.611	0.727
GBM	0.735	0.596	0.666	0.582	0.771	0.66
Neural Network	0.727	0.487	0.693	0.786	0.576	0.741
Random Forest	0.737	0.583	0.675	0.654	0.701	0.692
KNN	0.706	0.537	0.672	0.731	0.597	0.713
Adaboost	0.723	0.587	0.666	0.632	0.708	0.678
LightGBM	0.739	0.537	0.69	0.824	0.521	0.748
CatBoost	0.75	0.498	0.712	0.841	0.549	0.765

In the training set (Table 3), all models achieved an AUC > 0.73. The random forest model yielded the highest AUC (0.978) and sensitivity (0.920), but it may be prone to overfitting in real‐world applications. By contrast, CatBoost demonstrated a more balanced performance, with an AUC of 0.846, sensitivity of 0.838, accuracy of 0.782, and an F1 score of 0.811. Compared with LightGBM (AUC = 0.805) and KNN (AUC = 0.831), CatBoost showed superior overall performance, with a notably higher specificity (0.712 vs. 0.653 for LightGBM) and without the potential overfitting signal observed for the random forest model, indicating better discriminative ability and stability.

In the test set (Table 4), CatBoost maintained stable performance, achieving an AUC of 0.750, accuracy of 0.712, sensitivity of 0.841, and an F1 score of 0.765, outperforming all other models. Although LightGBM showed a slightly higher sensitivity (0.824), its lower specificity (0.521) resulted in inferior accuracy (0.690) compared with CatBoost. The AUC of the random forest model decreased to 0.737 in the test set, supporting the presence of overfitting in the training phase. The SVM model exhibited relatively higher specificity (0.611) but lower sensitivity (0.747). The neural network model achieved reasonable sensitivity (0.786) but underperformed in specificity and accuracy, yielding a lower F1 score than CatBoost (0.741 vs. 0.765). To further illustrate the differences in overall performance across models, radar chart comparisons of the top five best‐performing models are presented in Supporting Figure 1.

Across the external validation cohorts, model performance showed inter‐center heterogeneity. In the Qingdao cohort (Supporting Table 4), GBM had the highest AUC (0.741, 95% CI 0.601–0.882; F1 0.593); CatBoost yielded AUC 0.704 (95% CI 0.555–0.853) with sensitivity 0.696, while AdaBoost achieved the highest specificity (0.930) at the cost of sensitivity (0.522). In the Taian cohort (Supporting Table 5), GBM again led on AUC (0.792, 95% CI 0.652–0.932); CatBoost achieved AUC 0.729 (95% CI 0.573–0.886) with specificity 0.833, accuracy 0.643, and F1 score 0.615—better overall classification than LightGBM (AUC 0.729, specificity 0.722, accuracy 0.619) at the same discrimination level.

To assess whether the external validation results were affected by the imputation strategy, this study further conducted a sensitivity analysis using simple imputation based on summary statistics from each external validation cohort. The AUC changed from 0.704 to 0.7025 in the Qingdao cohort (Δ = 0.0015) and from 0.7292 to 0.7176 in the Taian cohort (Δ = 0.0116), suggesting that the external validation performance of the model was not substantially affected by the imputation strategy. Detailed performance metrics and AUC curves for each model after simple imputation are shown in Supporting Tables 15 and 16 and Supporting Figure 3.

Across both external cohorts, CatBoost maintained acceptable discriminative performance (Qingdao AUC 0.704; Taian AUC 0.729) with consistently balanced metrics. Combined with its strong internal test set results (AUC 0.750; sensitivity 0.841; F1 0.765), CatBoost was selected as the primary model for SHAP‐based interpretation and clinical translation.

### 4.5 Model Interpretation

To further elucidate the predictive mechanism of the CatBoost model, this study applied SHAP to provide both global and individual‐level interpretations.

#### 4.5.1 Global Feature Importance

**Figure 5:**
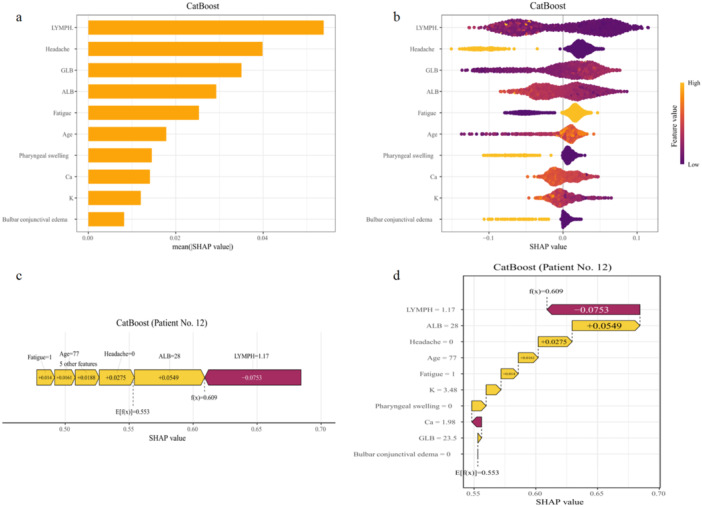
SHAP‐based interpretation of the CatBoost model. (a) Global feature importance ranked by mean absolute SHAP values; LYMPH# was the most influential predictor. (b) SHAP summary plot showing feature effect distribution; each dot represents a patient, color indicates feature value (yellow = high, purple = low). (c) SHAP force plot for Patient No. 12; yellow segments increase risk, purple segments decrease risk (E[f(x)] = 0.553 → f(x) = 0.609). (d) SHAP waterfall plot for Patient No. 12 showing stepwise feature contributions. SHAP SHapley Additive exPlanations, E[f(x)] Expected (baseline) prediction, f(x) Final predicted probability.

SHAP analysis quantified the relative importance of the ten predictors for the risk of myocardial injury in SFTS (Figure 5a). LYMPH# was the most influential predictor (mean absolute SHA *p* value = 0.0538), markedly exceeding the other features, followed by headache (0.0399), globulin (0.0350), and albumin (0.0293). Predictors of moderate importance included fatigue (0.0253) and age (0.0178), whereas pharyngeal swelling (0.0145), serum calcium (0.0141), serum potassium (0.0120), and bulbar conjunctival edema (0.0082) contributed relatively less. Detailed mean SHAP values are provided in Supporting Table 6.

The SHAP summary beeswarm plot (Figure 5b) further illustrated the direction and distribution of each feature's effect on the predicted risk of myocardial injury. The x‐axis represents the SHAP value (positive values increase the predicted probability; negative values decrease it); color indicates feature magnitude (yellow = higher; purple = lower). Among laboratory variables, lower LYMPH#, lower ALB, lower Ca, and lower K were each associated with increased predicted myocardial injury risk, whereas higher GLB was also positively associated with risk. Among clinical features, the presence of fatigue increased the predicted probability. Patients without headache, without pharyngeal swelling, and without bulbar conjunctival edema were predicted to have higher risk—consistent with the baseline finding that these symptoms were more frequent in the non–myocardial injury group—suggesting that the absence of local manifestations may reflect progression to a more severe disease stage. Older age was a positive predictor throughout.

#### 4.5.2 Individual‐Level Explanation

To illustrate individual‐level interpretability, this study visualized the prediction for Patient 12 using SHAP waterfall and force plots. The patient was 77 years old and presented with fatigue but without headache, pharyngeal swelling, or bulbar conjunctival edema. Admission laboratory values were: LYMPH# 1.17 × 10^9^/L, ALB 28 g/L, K 3.48 mmol/L, Ca 1.98 mmol/L, and GLB 23.5 g/L. The patient subsequently developed SFTS‐associated myocardial injury.

The force plot (Figure 5c) provides an intuitive visualization of how each feature contributed to the predicted probability of myocardial injury for this patient. Compared with the model's baseline expected prediction (E[f(x)] = 0.553), the patient's predicted probability was 0.609. Low albumin was the largest positive contributor (+0.0549), followed by absence of headache (+0.0275), older age (+0.0161), and fatigue (+0.0140 SHAP units). In contrast, a relatively higher lymphocyte count exerted a substantial negative contribution (−0.0753).

The waterfall plot (Figure 5d) traces the stepwise accumulation from baseline 0.553 to the final predicted value of 0.609: higher GLB, lower Ca, absence of pharyngeal swelling, lower K, fatigue, older age, absence of headache, and low ALB each contributed positively, while the lymphocyte count pulled the estimate downward (−0.0753).

This case‐level analysis identifies low albumin as the dominant risk driver, partially offset by a relatively preserved lymphocyte count. Such individual‐level interpretation not only enhances model transparency but also provides clinically actionable guidance: for high‐risk patients, clinicians should monitor albumin and lymphocyte count closely and consider early intervention when these values cross clinical thresholds.

### 4.6 Development of Clinical Decision Tool

**Figure 6:**
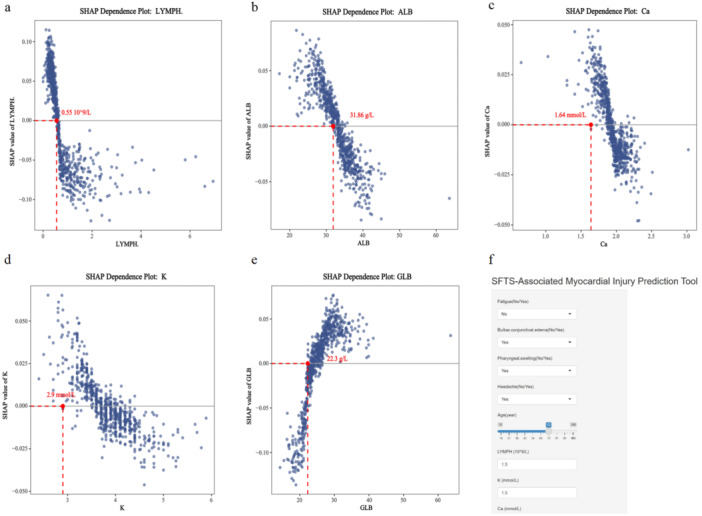
SHAP dependence plots and clinical decision tool for SFTS‐associated myocardial injury prediction. (a–e) SHAP dependence plots for five laboratory predictors. Red dashed lines indicate clinical risk thresholds: (a) LYMPH# < 0.55 × 10^9^/L; (b) ALB < 31.86 g/L; (c) Ca < 1.64 mmol/L; (d) K < 2.9 mmol/L; (e) GLB > 22.3 g/L. (f) Web‐based clinical prediction tool for real‐time risk calculation (0%–100%) based on 10 predictors.

To enhance the clinical translational value of the CatBoost model, SHAP dependence plots were used to quantify the associations between key laboratory indicators and the risk of myocardial injury (Figure 6a–e). The results showed that when lymphocyte count fell below 0.55 × 10^9^/L, albumin below 31.86 g/L, calcium below 1.64 mmol/L, potassium below 2.9 mmol/L, or globulin exceeded 22.3 g/L, the SHAP contribution shifted from negative to positive, indicating an increased probability of myocardial injury.

In addition, A web‐based clinical prediction tool was developed based on the CatBoost model (available at: https://lixiang2222.shinyapps.io/make_web/; a screenshot of the interface is shown in Figure 6f). By selecting, adjusting sliders, and entering values for the ten predictors, the tool provides real‐time estimation of the probability of incident myocardial injury in patients with SFTS (0%–100%), thereby offering practical support for individualized treatment planning.

## 5 Discussion

This multicenter retrospective cohort study identified early predictors of incident myocardial injury in hospitalized patients with SFTS and developed an explainable CatBoost‐based ML model. The model achieved AUCs of 0.750, 0.704, and 0.729 in the internal test set and two independent external validation cohorts, respectively, while maintaining high sensitivity and acceptable specificity.

SHAP analysis indicated that lymphocyte count was the most influential predictor (mean absolute SHA *p* value = 0.0538), substantially exceeding the contributions of the other features. Previous studies have demonstrated that lymphopenia commonly occurs in patients with SFTS and is closely associated with adverse outcomes [16, 17, 18]. Lymphopenia not only reflects virus‐mediated immune injury but is also closely linked to the development of cytokine storm [19, 20]. In this context, a reduced lymphocyte count may indicate more severe systemic inflammation and immune dysregulation, thereby increasing the risk of myocardial injury.

Albumin and globulin, as important indicators reflecting nutritional status and inflammatory activity [21], both showed substantial predictive value in our study. Previous studies have shown that hypoalbuminemia is a key determinant of poor prognosis in patients with SFTS, potentially attributable to infection‐related hepatic dysfunction and increased vascular permeability [22, 23]. Accordingly, it is hypothesize that hypoalbuminemia may increase the risk of myocardial injury through at least two pathways. First, low albumin levels often accompany persistent systemic inflammation [24], and inflammatory mediators may directly injure cardiomyocytes. Second, reduced plasma colloid osmotic pressure may promote myocardial interstitial edema, thereby impairing myocardial perfusion and energy metabolism. Moreover, SHAP dependence analysis in our study showed a marked increase in the predicted risk of myocardial injury when albumin levels fell below 31.86 g/L, providing a quantitative threshold to inform early clinical intervention. Conversely, higher globulin levels were positively associated with the risk of myocardial injury, which may reflect a persistently activated inflammatory state in patients with SFTS [25].

Electrolyte disturbances are common metabolic abnormalities in patients with SFTS. In this study, both hypocalcemia and hypokalemia were associated with an increased risk of myocardial injury. Previous studies have shown that calcium ions play a central role in excitation–contraction coupling in cardiomyocytes, and hypocalcemia may lead to reduced myocardial contractility and arrhythmias [26, 27]. Potassium is essential for maintaining the stability of the cardiomyocyte membrane potential, and hypokalemia may trigger arrhythmias, thereby increasing the risk of myocardial injury [28, 29]. The specific thresholds identified—Ca < 1.64 mmol/L and K < 2.9 mmol/L—may guide clinical monitoring and timely electrolyte correction.

Notably, the results showed that patients without headache, pharyngeal swelling, or bulbar conjunctival edema were at a higher risk of myocardial injury. It is speculated that the absence of these local manifestations may indicate progression to a more severe disease stage, in which systemic inflammatory responses and organ injury become the predominant clinical features, whereas early local immune‐response–related symptoms are less prominent. Clinically, this observation underscores the need for heightened vigilance in patients who lack typical early SFTS manifestations.

Older age was confirmed as a positive predictor in our study, consistent with previous reports [14, 15]. This association may be attributable to immunosenescence in older adults, leading to impaired ability to resist and clear SFTS virus, which in turn may predispose them to cytokine storm and subsequent myocardial injury. Fatigue, one of the most common clinical manifestations of SFTS [30], may increase the predicted probability of myocardial injury for several reasons. Fatigue often reflects impaired functional status and a heightened systemic inflammatory burden [31]. Persistent systemic inflammation and hypoperfusion may in turn affect the myocardium, thereby increasing the risk of myocardial injury.

Compared with traditional statistical approaches, machine learning algorithms offer distinct advantages in modeling high‐dimensional data and capturing nonlinear relationships^9^. In this study, LASSO regression and the Boruta algorithm were applied for feature selection, which effectively reduced redundant variables and mitigated multicollinearity. CatBoost offers distinct advantages in handling categorical features and missing data and can effectively reduce the risk of overfitting [32]. The use of SHAP not only quantified the contribution of each feature but also enhanced the transparency of the model's decision‐making process, which facilitates clinicians' understanding of and confidence in the rationale underlying the predictions.

This study has several limitations. First, the definition of myocardial injury relied on a center‐specific laboratory reference threshold (cTnI > 0.028 ng/mL). Because assay platforms and reagent sensitivities vary across institutions, this may limit the model's generalizability and transportability to other settings. Second, this was a retrospective study with a relatively long inclusion period (from May 2011 to October 2024), which may have introduced certain selection and information biases. In addition, subtle differences in clinical practice across different periods may have resulted in residual temporal confounding. Third, although external validation was performed in two independent cohorts, the sample sizes were relatively small (*n*1 = 80; *n*2 = 42), which may partly explain the modest performance decline and calibration drift (Supporting Figure 2) observed in the external validation sets. Nevertheless, the model maintained acceptable discriminative ability (AUC: 0.704 and 0.729) in both external cohorts, indicating that the model retains clinical utility for risk stratification. Moreover, both validation cohorts were from Shandong Province; therefore, further validation in populations from other regions is warranted. Fourth, only baseline admission variables were included, precluding assessment of how dynamic changes in predictors may influence the risk of myocardial injury. Finally, potentially important biological factors such as viral load and cytokine profiles were not incorporated, which may limit deeper mechanistic insights into myocardial injury in SFTS.

Prospective multicenter studies with standardized laboratory protocols and treatment strategies, inclusion of virological and immunological markers, integration of longitudinal clinical data, and randomized evaluation of model‐guided management strategies would all help to address these gaps.

## Author Contributions

Xiang Li conceptualized the study, performed the primary statistical modeling and clinical prediction analysis, and drafted the initial manuscript. Xiaotong Yu was responsible for the systematic collection and organization of clinical data and assisted in the initial drafting of the manuscript. Wei Zhou led the data processing work and undertook the inspection of multi‐center clinical data. Sujuan Zhang assisted in data interpretation and provided critical revisions to the manuscript. Zibo Fan led data analysis and took charge of manuscript drafting and revision. Yuanni Liu, Yi Shen, and Zhenghua Zhao collected and organized clinical data. Jianping Duan was responsible for the quality control of multi‐center data. Ling Lin oversaw the organization and analysis of data. Zhihai Chen supervised the multicenter study, provided clinical advice on SFTS‐associated complications, and coordinated data management. Wei Zhang supervised the entire research process, guided the interpretation of results and the formulation of core research ideas, and reviewed and finalized the manuscript.

## Ethics Statement

The research was conducted in accordance with the Declaration of Helsinki. Ethics approval was obtained from the Institutional Review Board of the Beijing Ditan Hospital, Capital Medical University (No. DTEC‐KY2022‐022‐02). As this retrospective analysis utilized routinely collected data, we submitted an application to our institutional review board for a waiver of informed consent, and it was granted by the Institutional Review Board of the Beijing Ditan Hospital, Capital Medical University.

## Conflicts of Interest

The authors declare no conflicts of interest.

## Supporting information


**Figure S1:** Feature selection and model performance comparison in Dataset 2 (internal cohort). (a) Correlation heatmap of LASSO‐selected variables; color represents Pearson correlation coefficients (blue = −1, red = +1). (b) Radar chart comparing top 5 models in the test set across five metrics (AUC, accuracy, F1, sensitivity, specificity); CatBoost (red) showed best performance. **Figure S2:** Calibration curves of machine learning models. Calibration curves showing the agreement between predicted probabilities and observed event percentages in (a) training set of Dataset 2, (b) testing set of Dataset 2, (c) Qingdao external validation set, and (d) Taian external validation set. The dashed diagonal line represents perfect calibration. **Figure S3:** Sensitivity analysis ROC curves in the two external validation cohorts (cohort‐internal simple imputation). (a–b) ROC curves of all nine machine learning models in the Qingdao and Taian cohorts, respectively. (c–d) ROC curves of the CatBoost model in the Qingdao and Taian cohorts.

## Data Availability

The data that support the findings of this study are available on request from the corresponding author. The data are not publicly available due to privacy or ethical restrictions. The data that support the findings of this study are available from the corresponding author upon reasonable request.
