# Supplementary material for: An Explainable Machine Learning Model for Early Prediction of Incident Myocardial Injury in Patients With Severe Fever With Thrombocytopenia Syndrome
Source: J Med Virol. 2026 Jun 29;98(7):e71044. doi: 10.1002/jmv.71044 (PMC13312896; doi:10.1002/jmv.71044)
Supplement: Supplementary file 1 — Figure S1: Feature selection and model performance comparison in Dataset 2 (internal cohort). (a) Correlation heatmap of LASSO‐selected variables; color represents Pearson correlation coefficients (blue = −1, red = +1). (b) Radar chart comparing top 5 models in the test set across five metrics (AUC, accuracy, F1, sensitivity, specificity); CatBoost (red) showed best performance. Figure S2: Calibration curves of machine learning models. Calibration curves showing the agreement between predicted probabilities and observed event percentages in (a) training set of Dataset 2, (b) testing set of Dataset 2, (c) Qingdao external validation set, and (d) Taian external validation set. The dashed diagonal line represents perfect calibration. Figure S3: Sensitivity analysis ROC curves in the two external validation cohorts (cohort‐internal simple imputation). (a–b) ROC curves of all nine machine learning models in the Qingdao and Taian cohorts, respectively. (c–d) ROC curves of the CatBoost model in the Qingdao and Taian cohorts. [file JMV-98-e71044-s001.docx]

Table S1 Optimized hyperparameters for each machine learning model

| **Model** | **Hyperparameter** |
| --- | --- |
| **Logistic Regression** | penalty=l2, C=1.0, max_iter=1000 |
| **SVM** | kernel:rbf,sigma:0.01,0.05,C:0.1,0.5 |
| **GBM** | n.trees:50,100,interaction.depth:1,2,shrinkage:0.01,0.05,n.minobsinnode:10,20 |
| **Neural Network** | size:1,2,decay:0.5,1 |
| **Random Forest** | mtry:2,3,ntree:150,nodesize:10 |
| **KNN** | kmax:35,45,55,distance:2,kernel:optimal |
| **AdaBoost** | mfinal:10,30,maxdepth:1,2,coeflearn:Zhu |
| **LightGBM** | objective:binary,metric:auc,learning_rate:0.03,max_depth:4,num_leaves:15,min_data_in_leaf:20,feature_fraction:0.8,bagging_fraction:0.8,bagging_freq:5,lambda_L1:0.5,lambda_L2:0.5,nrounds:200,early_stopping_rounds:20 |
| **CatBoost** | iterations:500,learning_rate:0.03,depth:6,l2_leaf_reg:10,bagging_temperature:0.5,random_strength:1,border_count:128,min_data_in_leaf:5,od_type:Iter,od_wait:50,loss_function:Logloss,eval_metric:AUC,task_type:CPU,verbose:50 |

Table S2 Baseline characteristics comparison of External queue (Qindao) between Myocardial Injury and Non-Myocardial Injury

| **Characteristics** | **Overall**  **（N＝80）** | **Non-Myocardial-Injury**  **（N＝57）** | **Myocardial-Injury**  **（N＝23）** | ***P* Value** |
| --- | --- | --- | --- | --- |
| **General information,**  **median (IQR) or n (%)** |  |  |  |  |
| Age (years) | 63.50 [54.50, 71.00] | 60.00 [53.00, 71.00] | 65.00 [57.50, 71.50] | 0.312 |
| Gender |  |  |  | 0.203 |
| Female | 38 (47.5) | 24 (42.1) | 14 (60.9) |  |
| Male | 42 (52.5) | 33 (57.9) | 9 (39.1) |  |
| **History of underlying disease,**  **n (%)** |  |  |  |  |
| Hypertension | 16 (20.0) | 13 (22.8) | 3 (13.0) | 0.497 |
| Diabetes | 13 (16.2) | 10 (17.5) | 3 (13.0) | 0.874 |
| Cerebral infarction | 2 (2.5) | 2 (3.5) | 0 (0.0) | 0.906 |
| **Symptoms at admission, n (%)** |  |  |  |  |
| Fever | 66 (82.5) | 45 (78.9) | 21 (91.3) | 0.321 |
| Fatigue | 73 (91.2) | 50 (87.7) | 23 (100.0) | 0.186 |
| Lethargy | 52 (68.4) | 34 (63.0) | 18 (81.8) | 0.183 |
| Headache | 17 (24.3) | 10 (20.0) | 7 (35.0) | 0.311 |
| Palpitation | 3 (4.2) | 3 (5.8) | 0 (0.0) | 0.687 |
| Muscle soreness | 22 (29.7) | 11 (21.2) | 11 (50.0) | 0.028 |
| Arthralgia | 11 (15.5) | 8 (15.7) | 3 (15.0) | 1 |
| Anorexia | 69 (87.3) | 50 (87.7) | 19 (86.4) | 1 |
| Nausea | 25 (34.7) | 17 (32.7) | 8 (40.0) | 0.759 |
| Vomiting | 17 (23.9) | 12 (23.5) | 5 (25.0) | 1 |
| Abdominal pain | 6 (8.6) | 3 (6.0) | 3 (15.0) | 0.458 |
| Diarrhea | 16 (22.5) | 12 (23.5) | 4 (20.0) | 0.996 |
| Cough | 11 (15.3) | 8 (15.1) | 3 (15.8) | 1 |
| Oliguria | 4 (5.7) | 3 (6.0) | 1 (5.0) | 1 |
| Cutaneous congestion | 7 (9.7) | 6 (11.5) | 1 (5.0) | 0.693 |
| Rash | 5 (7.1) | 3 (6.0) | 2 (10.0) | 0.942 |
| Petechia | 8 (11.3) | 6 (11.8) | 2 (10.0) | 1 |
| Pharyngeal swelling | 32 (42.1) | 29 (51.8) | 3 (15.0) | 0.009 |
| Bulbar conjunctival edema | 18 (25.0) | 13 (25.5) | 5 (23.8) | 1 |
| Lymphadenopathy | 5 (6.9) | 2 (3.9) | 3 (14.3) | 0.288 |
| Hemorrhage | 3 (4.2) | 2 (3.9) | 1 (5.0) | 1 |
| Bilateral renal percussion tenderness | 2 (2.8) | 2 (3.8) | 0 (0.0) | 0.929 |
| **Laboratory variables,**  **median (IQR)** |  |  |  |  |
| WBC(10^9/L) | 2.83 [1.84, 4.56] | 2.88 [2.04, 4.71] | 2.50 [1.71, 4.29] | 0.401 |
| NEUT#(10^9/L) | 1.55 [0.91, 2.91] | 1.51 [0.86, 2.75] | 1.73 [1.07, 3.46] | 0.566 |
| LYMPH# (10^9/L) | 0.78 [0.50, 1.36] | 1.01 [0.52, 1.48] | 0.51 [0.42, 0.76] | 0.008 |
| MONO#(10^9/L) | 0.19 [0.11, 0.38] | 0.22 [0.13, 0.43] | 0.10 [0.07, 0.28] | 0.012 |
| EO#(10^9/L) | 0.00 [0.00, 0.00] | 0.00 [0.00, 0.00] | 0.00 [0.00, 0.00] | 0.043 |
| BASO#(10^9/L) | 0.01 [0.00, 0.01] | 0.01 [0.00, 0.02] | 0.00 [0.00, 0.01] | 0.021 |
| RBC(10^12/L) | 4.56 [4.18, 4.90] | 4.58 [4.18, 5.01] | 4.43 [4.20, 4.84] | 0.303 |
| Hb(g/L) | 140.00 [126.00, 148.00] | 140.50 [127.75, 151.00] | 137.00 [125.50, 142.50] | 0.141 |
| MCV(fl) | 88.40 [86.05, 90.45] | 88.75 [87.23, 90.50] | 87.20 [84.45, 90.30] | 0.128 |
| MCHC (g/L) | 346.00 [339.00, 353.00] | 345.50 [339.00, 353.00] | 348.00 [338.00, 352.75] | 0.916 |
| RDW (%) | 12.90 [12.50, 13.55] | 12.80 [12.40, 13.50] | 13.00 [12.55, 13.70] | 0.32 |
| PLT (10^9/L) | 48.00 [35.75, 61.00] | 48.00 [37.00, 72.00] | 48.00 [30.00, 54.00] | 0.159 |
| MPV (fl) | 11.30 [10.50, 12.40] | 11.20 [10.50, 12.23] | 11.65 [10.00, 12.53] | 0.609 |
| K (mmol/L) | 3.70 [3.40, 4.10] | 3.70 [3.40, 4.05] | 3.70 [3.50, 4.07] | 0.959 |
| Na (mmol/L) | 135.00 [131.90, 138.00] | 135.00 [132.00, 138.75] | 133.05 [131.22, 136.75] | 0.466 |
| Cl (mmol/L) | 100.00 [97.00, 105.25] | 100.00 [96.25, 104.00] | 103.00 [98.00, 105.75] | 0.59 |
| Ca (mmol/L) | 1.92 [1.81, 2.07] | 1.96 [1.85, 2.07] | 1.82 [1.74, 1.89] | 0.047 |
| BUN (mmol/L) | 4.70 [3.40, 6.50] | 4.70 [3.68, 5.98] | 4.70 [3.30, 7.70] | 0.818 |
| Cr (μmol/L) | 67.00 [49.50, 93.50] | 73.00 [49.50, 93.50] | 60.50 [49.75, 95.75] | 0.99 |
| PT(s) | 11.10 [10.50, 11.70] | 11.05 [10.40, 11.70] | 11.10 [10.80, 11.80] | 0.405 |
| PTA (%) | 93.00 [87.00, 103.00] | 93.00 [85.00, 103.50] | 93.00 [88.04, 99.00] | 0.915 |
| APTT (s) | 39.80 [34.75, 43.98] | 39.10 [35.00, 43.35] | 41.70 [33.50, 46.45] | 0.669 |
| TT(s) | 18.30 [16.62, 19.88] | 18.30 [16.67, 19.88] | 17.90 [16.62, 19.88] | 0.951 |
| ALT (U/L) | 89.00 [52.00, 189.00] | 87.00 [59.00, 207.00] | 96.00 [45.00, 132.00] | 0.572 |
| AST (U/L) | 144.00 [89.00, 342.00] | 136.00 [89.00, 294.00] | 164.50 [94.00, 369.25] | 0.581 |
| TBIL (μmol/L) | 10.20 [8.03, 15.38] | 10.65 [8.47, 16.05] | 7.85 [6.69, 12.25] | 0.009 |
| DBIL (μmol/L) | 3.50 [2.62, 5.60] | 3.45 [2.50, 6.49] | 3.70 [2.74, 4.50] | 0.393 |
| TP (g/L) | 56.75 [52.50, 61.00] | 56.95 [52.10, 62.20] | 55.40 [53.02, 60.42] | 0.604 |
| ALB (g/L) | 31.10 [29.00, 34.38] | 33.10 [29.50, 35.00] | 29.85 [28.00, 32.60] | 0.021 |
| GLB (g/L) | 25.00 [21.10, 30.00] | 24.15 [21.08, 28.88] | 26.00 [21.50, 30.00] | 0.404 |
| GGT (U/L) | 39.00 [23.50, 67.00] | 40.00 [21.00, 82.00] | 37.50 [30.00, 52.00] | 0.973 |
| ALP (U/L) | 65.00 [54.00, 78.00] | 65.00 [55.50, 90.50] | 67.50 [55.50, 74.75] | 0.728 |
| CHE (U/L) | 5930.00 [4441.00, 7095.00] | 5972.00 [4647.50, 7607.25] | 5750.00 [4261.00, 6907.00] | 0.383 |

Table S3 Baseline characteristics comparison of External queue (Taian) between Myocardial Injury and Non-Myocardial Injury

| **Characteristics** | **Overall**  **（N＝42）** | **Non-Myocardial-Injury**  **（N＝18）** | **Myocardial-Injury**  **（N＝24）** | ***P* Value** |
| --- | --- | --- | --- | --- |
| **General information, median (IQR) or n (%)** |  |  |  |  |
| Age (years) | 59.00 [52.50, 66.75] | 58.00 [54.25, 62.75] | 61.50 [51.00, 68.50] | 0.38 |
| Gender |  |  |  | 1 |
| Female | 22 (52.4) | 9 (50.0) | 13 (54.2) |  |
| Male | 20 (47.6) | 9 (50.0) | 11 (45.8) |  |
| **History of underlying disease,**  **n (%)** |  |  |  |  |
| Hypertension | 8 (19.0) | 2 (11.1) | 6 (25.0) | 0.461 |
| Diabetes | 1 (2.4) | 0 (0.0) | 1 (4.2) | 1 |
| Cerebral infarction | 2 (4.8) | 0 (0.0) | 2 (8.3) | 0.601 |
| **Symptoms at admission, n (%)** |  |  |  |  |
| Fever | 30 (71.4) | 13 (72.2) | 17 (70.8) | 1 |
| Fatigue | 23 (65.7) | 11 (73.3) | 12 (60.0) | 0.644 |
| Lethargy | 18 (52.9) | 9 (60.0) | 9 (47.4) | 0.699 |
| Headache | 13 (37.1) | 8 (57.1) | 5 (23.8) | 0.101 |
| Muscle soreness | 10 (29.4) | 6 (46.2) | 4 (19.0) | 0.194 |
| Arthralgia | 8 (23.5) | 4 (30.8) | 4 (19.0) | 0.714 |
| Anorexia | 28 (82.4) | 12 (92.3) | 16 (76.2) | 0.462 |
| Nausea | 18 (50.0) | 8 (57.1) | 10 (45.5) | 0.732 |
| Vomiting | 11 (37.9) | 6 (46.2) | 5 (31.2) | 0.661 |
| Abdominal pain | 5 (14.3) | 3 (21.4) | 2 (9.5) | 0.622 |
| Diarrhea | 8 (22.9) | 4 (28.6) | 4 (19.0) | 0.805 |
| Cough | 4 (11.4) | 1 (7.7) | 3 (13.6) | 1 |
| Oliguria | 1 (2.9) | 0 (0.0) | 1 (4.8) | 1 |
| Cutaneous congestion | 7 (20.0) | 3 (23.1) | 4 (18.2) | 1 |
| Rash | 4 (11.1) | 3 (21.4) | 1 (4.5) | 0.304 |
| Pharyngeal swelling | 13 (36.1) | 6 (46.2) | 7 (30.4) | 0.561 |
| Bulbar conjunctival edema | 2 (6.9) | 0 (0.0) | 2 (11.8) | 0.626 |
| Lymphadenopathy | 12 (32.4) | 6 (40.0) | 6 (27.3) | 0.65 |
| Hemorrhage | 1 (2.8) | 0 (0.0) | 1 (4.5) | 1 |
| Bilateral renal percussion tenderness | 1 (2.9) | 0 (0.0) | 1 (4.5) | 1 |
| **Laboratory variables,**  **median (IQR)** |  |  |  |  |
| WBC(10^9/L) | 1.62 [1.17, 2.34] | 1.88 [1.51, 2.34] | 1.42 [1.11, 2.01] | 0.096 |
| NEUT#(10^9/L) | 0.95 [0.65, 1.41] | 1.19 [0.75, 1.60] | 0.82 [0.54, 1.20] | 0.16 |
| LYMPH# (10^9/L) | 0.42 [0.30, 0.90] | 0.46 [0.32, 1.06] | 0.42 [0.29, 0.66] | 0.4 |
| MONO#(10^9/L) | 0.08 [0.06, 0.16] | 0.10 [0.06, 0.25] | 0.08 [0.06, 0.10] | 0.323 |
| EO#(10^9/L) | 0.00 [0.00, 0.00] | 0.00 [0.00, 0.00] | 0.00 [0.00, 0.00] | 0.721 |
| BASO#(10^9/L) | 0.00 [0.00, 0.00] | 0.00 [0.00, 0.00] | 0.00 [0.00, 0.00] | 0.418 |
| RBC(10^12/L) | 4.44 [4.16, 4.65] | 4.43 [4.19, 4.72] | 4.44 [4.14, 4.64] | 0.703 |
| Hb(g/L) | 135.00 [127.00, 143.00] | 136.00 [128.75, 142.75] | 132.00 [126.00, 142.50] | 0.446 |
| MCV(fl) | 88.50 [85.60, 91.60] | 88.45 [86.02, 91.27] | 88.80 [86.00, 91.25] | 0.773 |
| MCHC (g/L) | 344.00 [339.00, 354.00] | 343.00 [339.00, 357.75] | 347.00 [339.50, 352.00] | 0.927 |
| RDW (%) | 14.90 [12.40, 40.30] | 13.00 [12.20, 37.30] | 38.30 [12.93, 40.83] | 0.093 |
| PLT (10^9/L) | 68.50 [51.50, 73.00] | 71.50 [64.50, 73.00] | 67.50 [42.50, 72.50] | 0.28 |
| MPV (fl) | 11.40 [10.50, 11.90] | 10.85 [10.43, 11.47] | 11.60 [11.00, 12.05] | 0.07 |
| K (mmol/L) | 3.62 [3.45, 4.11] | 3.63 [3.43, 4.06] | 3.62 [3.46, 4.11] | 0.817 |
| Na (mmol/L) | 134.20 [129.82, 137.15] | 134.95 [133.07, 138.65] | 133.25 [128.25, 136.00] | 0.075 |
| Cl (mmol/L) | 98.35 [95.40, 101.62] | 99.60 [97.55, 101.33] | 97.40 [93.00, 101.55] | 0.191 |
| Ca (mmol/L) | 1.98 [1.92, 2.08] | 2.05 [2.01, 2.14] | 1.92 [1.85, 1.97] | 0.001 |
| BUN (mmol/L) | 4.80 [3.75, 6.80] | 4.78 [3.17, 6.10] | 4.80 [3.80, 7.00] | 0.517 |
| Cr (μmol/L) | 70.00 [60.00, 84.00] | 70.00 [60.00, 95.00] | 70.00 [60.00, 79.00] | 0.895 |
| PT(s) | 12.55 [12.30, 13.40] | 12.55 [12.33, 13.30] | 12.60 [12.30, 13.40] | 0.939 |
| PTA (%) | 88.00 [78.95, 95.75] | 89.00 [75.00, 98.33] | 87.75 [80.53, 93.80] | 0.732 |
| APTT (s) | 39.25 [36.00, 45.10] | 37.80 [34.73, 39.27] | 41.35 [37.15, 47.22] | 0.077 |
| TT(s) | 18.30 [17.25, 19.35] | 17.55 [17.02, 19.08] | 18.50 [17.90, 19.90] | 0.108 |
| ALT (U/L) | 78.00 [39.00, 123.50] | 56.00 [27.00, 119.00] | 86.00 [45.00, 125.00] | 0.22 |
| AST (U/L) | 156.50 [77.75, 264.25] | 115.00 [63.25, 230.00] | 179.50 [121.50, 288.00] | 0.236 |
| TBIL (μmol/L) | 6.80 [4.90, 9.57] | 7.10 [5.95, 9.22] | 5.80 [4.80, 10.10] | 0.672 |
| DBIL (μmol/L) | 3.75 [2.95, 5.00] | 3.85 [3.52, 4.85] | 3.50 [2.60, 5.20] | 0.671 |
| TP (g/L) | 59.30 [54.80, 63.35] | 59.70 [57.33, 64.92] | 58.40 [54.65, 61.92] | 0.293 |
| ALB (g/L) | 35.20 [32.25, 38.05] | 37.70 [34.07, 40.72] | 32.75 [31.20, 36.47] | 0.008 |
| GLB (g/L) | 23.45 [21.35, 25.95] | 22.25 [21.12, 25.78] | 24.00 [22.83, 27.77] | 0.179 |
| GGT (U/L) | 31.00 [22.00, 70.00] | 26.50 [19.75, 57.25] | 42.00 [22.50, 87.50] | 0.287 |
| ALP (U/L) | 62.50 [53.00, 72.50] | 65.50 [53.75, 72.50] | 58.50 [53.00, 69.00] | 0.65 |

Table S4 Performance comparison of different machine learning models in the external validation set 1 (Qindao)

| **Model** | **AUC** | **Threshold** | **Sensitivity** | **Specificity** | **Accuracy** | **F1 Score** |
| --- | --- | --- | --- | --- | --- | --- |
| GBM | 0.7414 | 0.5429 | 0.6957 | 0.7368 | 0.725 | 0.5926 |
| Adaboost | 0.7216 | 0.62 | 0.5217 | 0.9298 | 0.8125 | 0.6154 |
| Neural Network | 0.7185 | 0.5589 | 0.6087 | 0.7193 | 0.6875 | 0.5283 |
| Logistic | 0.7056 | 0.6478 | 0.5217 | 0.8421 | 0.75 | 0.5455 |
| CatBoost | 0.704 | 0.5272 | 0.6957 | 0.5263 | 0.575 | 0.4848 |
| LightGBM | 0.6876 | 0.552 | 0.7826 | 0.4912 | 0.575 | 0.5143 |
| SVM | 0.6873 | 0.585 | 0.4783 | 0.7719 | 0.6875 | 0.4681 |
| Random Forest | 0.6621 | 0.57 | 0.6087 | 0.6316 | 0.625 | 0.4828 |
| KNN | 0.5736 | 0.535 | 0.5652 | 0.5263 | 0.5375 | 0.4127 |

Table S5 Performance comparison of different machine learning models in the external validation set 2 (Taian)

| Model | AUC | Threshold | Sensitivity | Specificity | Accuracy | F1 Score |
| --- | --- | --- | --- | --- | --- | --- |
| GBM | 0.7917 | 0.5429 | 0.5833 | 0.8889 | 0.7143 | 0.7 |
| Adaboost | 0.7431 | 0.62 | 0.3333 | 0.8889 | 0.5714 | 0.4706 |
| Random Forest | 0.7407 | 0.57 | 0.4583 | 0.8889 | 0.6429 | 0.5946 |
| Neural Network | 0.7338 | 0.5589 | 0.4583 | 0.8889 | 0.6429 | 0.5946 |
| Logistic | 0.7292 | 0.6478 | 0.3333 | 1 | 0.619 | 0.5 |
| LightGBM | 0.7292 | 0.552 | 0.5417 | 0.7222 | 0.619 | 0.619 |
| CatBoost | 0.7292 | 0.5272 | 0.5 | 0.8333 | 0.6429 | 0.6154 |
| SVM | 0.6991 | 0.585 | 0.375 | 0.8889 | 0.5952 | 0.5143 |
| KNN | 0.6829 | 0.535 | 0.4583 | 0.7778 | 0.5952 | 0.5641 |

Table S6 SHAP-based Feature Importance for SFTS Myocardial Injury Prediction

| Rank | Feature | Mean SHAP Value |
| --- | --- | --- |
| 1 | LYMPH. | 0.0538 |
| 2 | Headache | 0.0399 |
| 3 | GLB | 0.035 |
| 4 | ALB | 0.0293 |
| 5 | Fatigue | 0.0253 |
| 6 | Age | 0.0178 |
| 7 | Pharyngeal swelling | 0.0145 |
| 8 | Ca | 0.0141 |
| 9 | K | 0.012 |
| 10 | Bulbar conjunctival edema | 0.0082 |

Table S7 Performance comparison of different machine learning models in the training set imp1

| Model | AUC | Threshold | Accuracy | Sensitivity | Specificity | F1 Score |
| --- | --- | --- | --- | --- | --- | --- |
| Logistic | 0.731 | 0.629 | 0.668 | 0.6 | 0.754 | 0.668 |
| SVM | 0.736 | 0.648 | 0.673 | 0.598 | 0.769 | 0.671 |
| GBM | 0.787 | 0.564 | 0.724 | 0.718 | 0.733 | 0.744 |
| NeuralNetwork | 0.733 | 0.582 | 0.697 | 0.696 | 0.697 | 0.719 |
| RandomForest | 0.972 | 0.537 | 0.924 | 0.934 | 0.911 | 0.932 |
| KNN | 0.849 | 0.537 | 0.77 | 0.828 | 0.697 | 0.801 |
| Adaboost | 0.751 | 0.567 | 0.705 | 0.744 | 0.656 | 0.737 |
| LightGBM | 0.808 | 0.548 | 0.748 | 0.812 | 0.668 | 0.782 |
| CatBoost | 0.85 | 0.547 | 0.781 | 0.814 | 0.739 | 0.806 |

Table S8 Performance comparison of different machine learning models in the test set imp1

| Model | AUC | Threshold | Accuracy | Sensitivity | Specificity | F1 Score |
| --- | --- | --- | --- | --- | --- | --- |
| Logistic | 0.709 | 0.52 | 0.684 | 0.775 | 0.569 | 0.732 |
| SVM | 0.716 | 0.551 | 0.687 | 0.769 | 0.583 | 0.733 |
| GBM | 0.735 | 0.502 | 0.696 | 0.78 | 0.59 | 0.742 |
| NeuralNetwork | 0.72 | 0.451 | 0.702 | 0.83 | 0.542 | 0.757 |
| RandomForest | 0.736 | 0.457 | 0.702 | 0.835 | 0.535 | 0.758 |
| KNN | 0.713 | 0.566 | 0.684 | 0.714 | 0.646 | 0.716 |
| Adaboost | 0.722 | 0.589 | 0.681 | 0.621 | 0.757 | 0.685 |
| LightGBM | 0.733 | 0.544 | 0.687 | 0.786 | 0.562 | 0.737 |
| CatBoost | 0.746 | 0.54 | 0.693 | 0.725 | 0.653 | 0.725 |

Table S9 Performance comparison of different machine learning models in the training set imp3

| Model | AUC | Threshold | Accuracy | Sensitivity | Specificity | F1 Score |
| --- | --- | --- | --- | --- | --- | --- |
| Logistic | 0.729 | 0.636 | 0.669 | 0.586 | 0.774 | 0.664 |
| SVM | 0.732 | 0.631 | 0.656 | 0.527 | 0.819 | 0.631 |
| GBM | 0.789 | 0.597 | 0.714 | 0.666 | 0.774 | 0.722 |
| NeuralNetwork | 0.745 | 0.614 | 0.696 | 0.645 | 0.76 | 0.703 |
| RandomForest | 0.973 | 0.55 | 0.92 | 0.941 | 0.893 | 0.929 |
| KNN | 0.825 | 0.52 | 0.755 | 0.835 | 0.653 | 0.792 |
| Adaboost | 0.75 | 0.557 | 0.703 | 0.727 | 0.674 | 0.732 |
| LightGBM | 0.802 | 0.571 | 0.735 | 0.736 | 0.733 | 0.756 |
| CatBoost | 0.848 | 0.535 | 0.781 | 0.835 | 0.712 | 0.81 |

Table S10 Performance comparison of different machine learning models in the test set imp3

| Model | AUC | Threshold | Accuracy | Sensitivity | Specificity | F1 Score |
| --- | --- | --- | --- | --- | --- | --- |
| Logistic | 0.713 | 0.503 | 0.684 | 0.802 | 0.535 | 0.739 |
| SVM | 0.723 | 0.482 | 0.687 | 0.764 | 0.59 | 0.732 |
| GBM | 0.736 | 0.496 | 0.702 | 0.808 | 0.569 | 0.752 |
| NeuralNetwork | 0.723 | 0.468 | 0.702 | 0.852 | 0.514 | 0.762 |
| RandomForest | 0.729 | 0.563 | 0.675 | 0.698 | 0.646 | 0.706 |
| KNN | 0.716 | 0.559 | 0.672 | 0.703 | 0.632 | 0.705 |
| Adaboost | 0.719 | 0.585 | 0.675 | 0.604 | 0.764 | 0.675 |
| LightGBM | 0.742 | 0.519 | 0.709 | 0.885 | 0.486 | 0.772 |
| CatBoost | 0.745 | 0.587 | 0.672 | 0.593 | 0.771 | 0.669 |

Table S11 Performance comparison of different machine learning models in the training set imp4

| Model | AUC | Threshold | Accuracy | Sensitivity | Specificity | F1 Score |
| --- | --- | --- | --- | --- | --- | --- |
| Logistic | 0.729 | 0.637 | 0.663 | 0.586 | 0.76 | 0.66 |
| SVM | 0.731 | 0.65 | 0.652 | 0.504 | 0.84 | 0.618 |
| GBM | 0.788 | 0.516 | 0.727 | 0.807 | 0.626 | 0.767 |
| NeuralNetwork | 0.739 | 0.608 | 0.677 | 0.626 | 0.742 | 0.684 |
| RandomForest | 0.975 | 0.517 | 0.92 | 0.936 | 0.899 | 0.929 |
| KNN | 0.83 | 0.557 | 0.753 | 0.786 | 0.712 | 0.78 |
| Adaboost | 0.754 | 0.568 | 0.707 | 0.685 | 0.736 | 0.723 |
| LightGBM | 0.81 | 0.553 | 0.757 | 0.795 | 0.709 | 0.785 |
| CatBoost | 0.845 | 0.545 | 0.774 | 0.793 | 0.751 | 0.797 |

Table S12 Performance comparison of different machine learning models in the test set imp4

| Model | AUC | Threshold | Accuracy | Sensitivity | Specificity | F1 Score |
| --- | --- | --- | --- | --- | --- | --- |
| Logistic | 0.702 | 0.545 | 0.663 | 0.714 | 0.597 | 0.703 |
| SVM | 0.717 | 0.525 | 0.681 | 0.714 | 0.639 | 0.714 |
| GBM | 0.724 | 0.512 | 0.696 | 0.775 | 0.597 | 0.74 |
| NeuralNetwork | 0.716 | 0.502 | 0.681 | 0.764 | 0.576 | 0.728 |
| RandomForest | 0.718 | 0.457 | 0.687 | 0.819 | 0.521 | 0.745 |
| KNN | 0.706 | 0.535 | 0.681 | 0.753 | 0.59 | 0.725 |
| Adaboost | 0.696 | 0.57 | 0.644 | 0.604 | 0.694 | 0.655 |
| LightGBM | 0.73 | 0.548 | 0.684 | 0.742 | 0.611 | 0.724 |
| CatBoost | 0.737 | 0.55 | 0.69 | 0.687 | 0.694 | 0.712 |

Table S13 Performance comparison of different machine learning models in the training set imp5

| Model | AUC | Threshold | Accuracy | Sensitivity | Specificity | F1 Score |
| --- | --- | --- | --- | --- | --- | --- |
| Logistic | 0.724 | 0.648 | 0.655 | 0.551 | 0.786 | 0.64 |
| SVM | 0.725 | 0.629 | 0.654 | 0.541 | 0.795 | 0.635 |
| GBM | 0.786 | 0.612 | 0.707 | 0.621 | 0.816 | 0.703 |
| NeuralNetwork | 0.735 | 0.575 | 0.685 | 0.668 | 0.706 | 0.703 |
| RandomForest | 0.976 | 0.537 | 0.929 | 0.946 | 0.908 | 0.937 |
| KNN | 0.803 | 0.548 | 0.731 | 0.798 | 0.647 | 0.768 |
| Adaboost | 0.728 | 0.623 | 0.684 | 0.746 | 0.605 | 0.725 |
| LightGBM | 0.804 | 0.574 | 0.732 | 0.734 | 0.73 | 0.754 |
| CatBoost | 0.843 | 0.535 | 0.778 | 0.84 | 0.7 | 0.809 |

Table S14 Performance comparison of different machine learning models in the test set imp5

| Model | AUC | Threshold | Accuracy | Sensitivity | Specificity | F1 Score |
| --- | --- | --- | --- | --- | --- | --- |
| Logistic | 0.702 | 0.542 | 0.669 | 0.709 | 0.618 | 0.705 |
| SVM | 0.717 | 0.476 | 0.681 | 0.769 | 0.569 | 0.729 |
| GBM | 0.72 | 0.536 | 0.672 | 0.742 | 0.583 | 0.716 |
| NeuralNetwork | 0.711 | 0.48 | 0.678 | 0.775 | 0.556 | 0.729 |
| RandomForest | 0.7 | 0.517 | 0.675 | 0.747 | 0.583 | 0.72 |
| KNN | 0.707 | 0.589 | 0.647 | 0.61 | 0.694 | 0.659 |
| Adaboost | 0.679 | 0.67 | 0.623 | 0.566 | 0.694 | 0.626 |
| LightGBM | 0.724 | 0.593 | 0.647 | 0.538 | 0.785 | 0.63 |
| CatBoost | 0.732 | 0.48 | 0.696 | 0.863 | 0.486 | 0.76 |

Table S15 Predictive performance of machine learning models in the Qingdao external validation cohort after simple imputation

| Model | AUC | Threshold | Sensitivity | Specificity | Accuracy | F1 Score |
| --- | --- | --- | --- | --- | --- | --- |
| GBM | 0.7429 | 0.5429 | 0.6957 | 0.7193 | 0.7125 | 0.5818 |
| Adaboost | 0.7311 | 0.62 | 0.5217 | 0.8246 | 0.7375 | 0.5333 |
| Neural Network | 0.7208 | 0.5589 | 0.6087 | 0.7193 | 0.6875 | 0.5283 |
| Logistic | 0.7063 | 0.6478 | 0.5217 | 0.8421 | 0.75 | 0.5455 |
| CatBoost | 0.7025 | 0.5272 | 0.6957 | 0.5263 | 0.575 | 0.4848 |
| LightGBM | 0.6922 | 0.552 | 0.7826 | 0.5088 | 0.5875 | 0.5217 |
| SVM | 0.688 | 0.585 | 0.4783 | 0.7719 | 0.6875 | 0.4681 |
| Random Forest | 0.6491 | 0.57 | 0.6087 | 0.5965 | 0.6 | 0.4667 |
| KNN | 0.569 | 0.535 | 0.5652 | 0.5088 | 0.525 | 0.4062 |

Table S16 Predictive performance of machine learning models in the Taian external validation cohort after simple imputation

| Model | AUC | Threshold | Sensitivity | Specificity | Accuracy | F1 Score |
| --- | --- | --- | --- | --- | --- | --- |
| GBM | 0.787 | 0.5429 | 0.5833 | 0.8889 | 0.7143 | 0.7 |
| Adaboost | 0.7384 | 0.62 | 0.375 | 0.8889 | 0.5952 | 0.5143 |
| Random Forest | 0.7269 | 0.57 | 0.4583 | 0.8889 | 0.6429 | 0.5946 |
| Neural Network | 0.7315 | 0.5589 | 0.4583 | 0.8889 | 0.6429 | 0.5946 |
| Logistic | 0.7245 | 0.6478 | 0.3333 | 1 | 0.619 | 0.5 |
| LightGBM | 0.7176 | 0.552 | 0.5417 | 0.7222 | 0.619 | 0.619 |
| CatBoost | 0.7176 | 0.5272 | 0.5 | 0.8333 | 0.6429 | 0.6154 |
| SVM | 0.6968 | 0.585 | 0.375 | 0.8889 | 0.5952 | 0.5143 |
| KNN | 0.6806 | 0.535 | 0.4583 | 0.7778 | 0.5952 | 0.5641 |

Table S17 Missing data summary and imputation strategy for variables in the internal cohort

| **Characteristics** | **Missing_Count** | **Total_Count** | **Missing_Rate (%)** | **Imputation_Strategy** |
| --- | --- | --- | --- | --- |
| APTT | 238 | 1088 | 21.88 | MICE Imputation (5%~30%) |
| PTA | 219 | 1088 | 20.13 | MICE Imputation (5%~30%) |
| TT | 187 | 1088 | 17.19 | MICE Imputation (5%~30%) |
| ALT | 175 | 1088 | 16.08 | MICE Imputation (5%~30%) |
| PT | 161 | 1088 | 14.8 | MICE Imputation (5%~30%) |
| GGT | 159 | 1088 | 14.61 | MICE Imputation (5%~30%) |
| CHE | 133 | 1088 | 12.22 | MICE Imputation (5%~30%) |
| Ca | 132 | 1088 | 12.13 | MICE Imputation (5%~30%) |
| DBIL | 115 | 1088 | 10.57 | MICE Imputation (5%~30%) |
| ALP | 111 | 1088 | 10.2 | MICE Imputation (5%~30%) |
| GLB | 110 | 1088 | 10.11 | MICE Imputation (5%~30%) |
| AST | 108 | 1088 | 9.93 | MICE Imputation (5%~30%) |
| TP | 107 | 1088 | 9.83 | MICE Imputation (5%~30%) |
| TBIL | 106 | 1088 | 9.74 | MICE Imputation (5%~30%) |
| ALB | 98 | 1088 | 9.01 | MICE Imputation (5%~30%) |
| BUN | 93 | 1088 | 8.55 | MICE Imputation (5%~30%) |
| Bulbar conjunctival edema | 89 | 1088 | 8.18 | MICE Imputation (5%~30%) |
| Cr | 84 | 1088 | 7.72 | MICE Imputation (5%~30%) |
| Vomiting | 70 | 1088 | 6.43 | MICE Imputation (5%~30%) |
| Lethargy | 65 | 1088 | 5.97 | MICE Imputation (5%~30%) |
| Fatigue | 63 | 1088 | 5.79 | MICE Imputation (5%~30%) |
| MPV | 56 | 1088 | 5.15 | MICE Imputation (5%~30%) |
| Cl | 55 | 1088 | 5.06 | MICE Imputation (5%~30%) |
| Petechia | 48 | 1088 | 4.41 | Simple Imputation (<5%) |
| Na | 47 | 1088 | 4.32 | Simple Imputation (<5%) |
| K | 46 | 1088 | 4.23 | Simple Imputation (<5%) |
| Palpitation | 45 | 1088 | 4.14 | Simple Imputation (<5%) |
| Hemorrhage | 45 | 1088 | 4.14 | Simple Imputation (<5%) |
| Arthralgia | 42 | 1088 | 3.86 | Simple Imputation (<5%) |
| Headache | 40 | 1088 | 3.68 | Simple Imputation (<5%) |
| Rash | 40 | 1088 | 3.68 | Simple Imputation (<5%) |
| Bilateral renal percussion tenderness | 40 | 1088 | 3.68 | Simple Imputation (<5%) |
| Muscle soreness | 39 | 1088 | 3.58 | Simple Imputation (<5%) |
| Abdominal pain | 37 | 1088 | 3.4 | Simple Imputation (<5%) |
| Diarrhea | 37 | 1088 | 3.4 | Simple Imputation (<5%) |
| Melena | 37 | 1088 | 3.4 | Simple Imputation (<5%) |
| Oliguria | 37 | 1088 | 3.4 | Simple Imputation (<5%) |
| Pharyngeal swelling | 37 | 1088 | 3.4 | Simple Imputation (<5%) |
| Lymphadenopathy | 37 | 1088 | 3.4 | Simple Imputation (<5%) |
| Cutaneous congestion | 35 | 1088 | 3.22 | Simple Imputation (<5%) |
| Cough | 34 | 1088 | 3.12 | Simple Imputation (<5%) |
| Nausea | 30 | 1088 | 2.76 | Simple Imputation (<5%) |
| Anorexia | 25 | 1088 | 2.3 | Simple Imputation (<5%) |
| RDW | 13 | 1088 | 1.19 | Simple Imputation (<5%) |
| MONO# | 6 | 1088 | 0.55 | Simple Imputation (<5%) |
| BASO# | 6 | 1088 | 0.55 | Simple Imputation (<5%) |
| RBC | 6 | 1088 | 0.55 | Simple Imputation (<5%) |
| Hb | 6 | 1088 | 0.55 | Simple Imputation (<5%) |
| NEUT# | 5 | 1088 | 0.46 | Simple Imputation (<5%) |
| MCHC | 5 | 1088 | 0.46 | Simple Imputation (<5%) |
| LYMPH# | 4 | 1088 | 0.37 | Simple Imputation (<5%) |
| EO# | 4 | 1088 | 0.37 | Simple Imputation (<5%) |
| MCV | 4 | 1088 | 0.37 | Simple Imputation (<5%) |
| WBC | 3 | 1088 | 0.28 | Simple Imputation (<5%) |
| Gender | 0 | 1088 | 0 | No Missing |
| Hypertension | 0 | 1088 | 0 | No Missing |
| Diabetes | 0 | 1088 | 0 | No Missing |
| Cerebral infarction | 0 | 1088 | 0 | No Missing |
| Fever | 0 | 1088 | 0 | No Missing |
| Age | 0 | 1088 | 0 | No Missing |
| PLT | 0 | 1088 | 0 | No Missing |


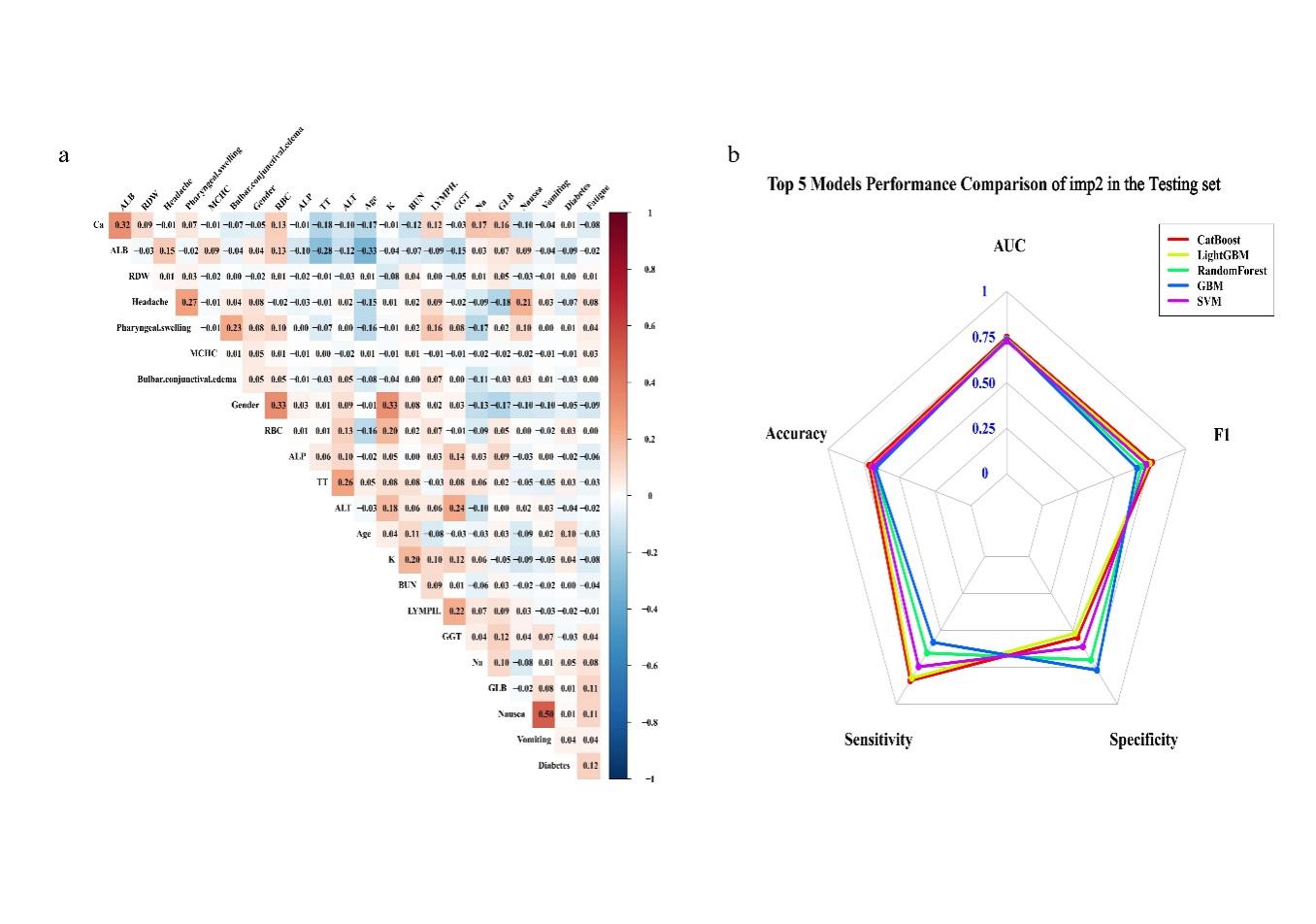


Figure S1 Feature selection and model performance comparison in Dataset 2 (internal cohort)


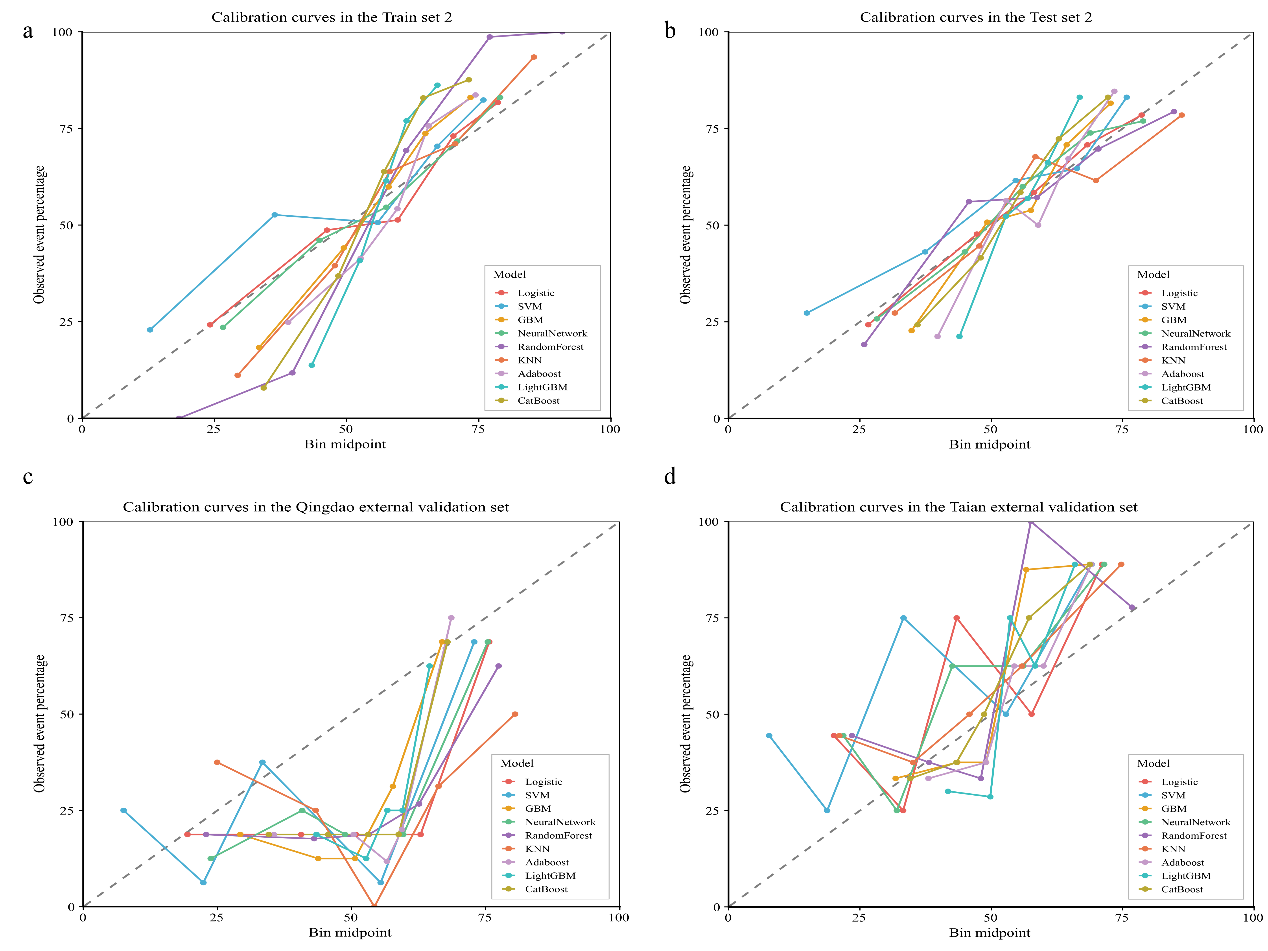
Figure S2 Calibration curves of machine learning models


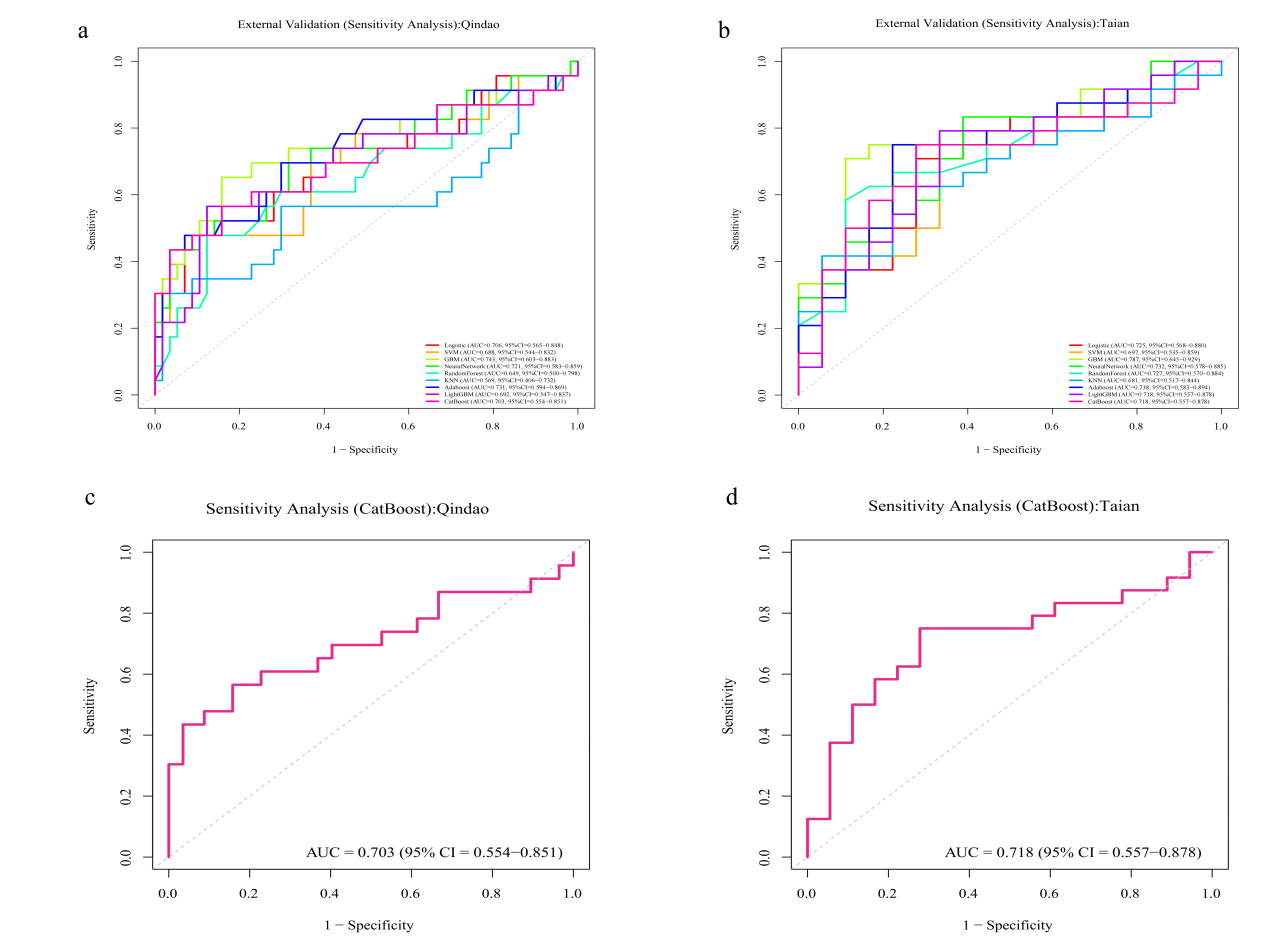


Figure S3 ROC curves of machine learning models in the external validation cohorts after simple imputation
